# Novel Isolongifolenone-Based Caprolactam Derivatives as Potential Anticancer Agents via the p53/mTOR/Autophagy Pathway

**DOI:** 10.3390/molecules30194013

**Published:** 2025-10-08

**Authors:** Yunyun Wang, Min Hu, Jiale Han, Yuxun Zhao, Biao Xiong, Peihai Li, Shifa Wang

**Affiliations:** 1 School of Pharmacy, Jiangsu Province Key Laboratory for Inflammation and Molecular Drug Target, Nantong University, Nantong 226001, China; 2323320001@stmail.ntu.edu.cn (M.H.); 2423320021@stmail.ntu.edu.cn (J.H.); hsiung1987@ntu.edu.cn (B.X.); 2 Co-Innovation Center of Efficient Processing and Utilization of Forest Resources, College of Chemical Engineering, Nanjing Forestry University, Nanjing 210037, China; 17600226827@163.com; 3 Engineering Research Center of Zebrafish Models for Human Diseases and Drug Screening of Shandong Province, Biology Institute, Qilu University of Technology (Shandong Academy of Sciences), Jinan 250103, China; lipeihaihg@sina.com

**Keywords:** isolongifolenone, caprolactam derivatives, antitumor activities, autophagy

## Abstract

Isolongifolenone, a natural sesquiterpenoid widely used in food additives and perfume, demonstrates a range of biological activities. In this study, a series of isolongifolenone-based caprolactam derivatives (**E1**–**E19**) were designed, synthesized, and evaluated for their anticancer activities in vitro. Most of the synthesized compounds significantly inhibited the proliferation of cultured cancer cells. Compound **E10**, containing an *m*-trifluoromethyl group, demonstrated the strongest anti-proliferation activities against MCF-7 (IC_50_ = 0.32 µM), HepG2 (IC_50_ = 1.36 µM), and A549 (IC_50_ = 1.39 µM) cells. Moreover, **E10** was shown to increase intracellular ROS, reduce mitochondrial function, and induce cancer cell apoptosis via the p53/mTOR/autophagy pathway. Together, these results indicate that compound **E10** induced autophagy-associated cell apoptosis in MCF-7 cancer cells. Additionally, the antitumor activity of **E10** was validated in a zebrafish MCF-7 xenograft model. The observation that **E10** exhibits potent antitumor activity in both a three-dimensional (3D) cell culture model and the zebrafish xenograft model supports the development of **E10** as a potential drug candidate for cancer therapy.

## 1. Introduction

Cancer remains one of the leading causes of human death [1]. Current first-line treatments for cancer include surgery, chemotherapy, and radiation. However, because tumor resection alone is typically ineffective (with remnant tumors commonly appearing in the resected tissue), combination therapies are used to enhance tumor treatment and prevent tumor recurrence. Chemotherapy is one of the most effective treatments for malignant tumors, and the clinical applications of antitumor drugs have markedly improved through better-dosing regimens, neoadjuvant or adjuvant therapy, and combination treatments. Despite these notable achievements in antitumor drug development, cancer remains a difficult medical challenge, particularly in the lungs, breast, liver, prostate, pancreas, and brain [2,3]. The primary causes for the failure of conventional cancer therapies (and the associated increase in cancers worldwide) are tumor metastasis and drug resistance. To effectively control and eradicate cancer in the future, there is a critical need to reevaluate current cancer therapy strategies in order to address these complications.

Natural sesquiterpenoids are valuable resources for organic and medicinal chemistry. Because of their peculiar structure, sesquiterpenoids have rapidly been adopted as a platform for obtaining biologically active compounds. To date, modification of natural products has proven to be a promising strategy to enhance their targeting properties and reduce their toxicities [4,5,6,7]. Isolongifolenone, a natural sesquiterpenoid obtained from turpentine, has been effectively applied in food additives and perfumes [8,9]. Despite their bulky structures, isolongifolenone sesquiterpenoids easily penetrate cell membranes, a property which determines both their bioavailability and a range of other biological properties. Recently, several studies have provided evidence that isolongifolenone and its derivatives exhibit versatile biological activities, including antioxidant activity [10], tyrosinase inhibitory activity [11], AMPK activator activity [12], RXR agonistic activity [13], and estrogen receptor inhibitor activity [14]. To date, structural modification of isolongifolenone has focused mainly on fusing it with nitrogen-containing heterocycles. In our earlier work, we reported the development of longifolene-derived nitrogen-containing heterocyclic derivatives with good bioactivities [15,16,17,18]. Therefore, the potential of isolongifolenone modification to generate derivatives with anticancer activities merits further exploration.

**Figure 1:**
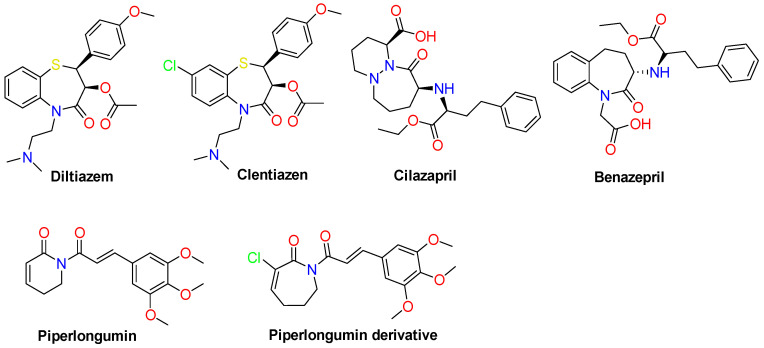
Chemical structures of Lactam derivatives.

Lactams, a class of cyclic amides, are prominent heterocycles within the medicinal chemistry and drug discovery fields. Compounds containing lactam moieties demonstrate a broad spectrum of biological activities, including antibacterial, antiviral, anticancer, anti-inflammatory, and neuroprotective effects on the central nervous system [19,20,21]. To take a classic example, *β*-lactam antibiotics [22,23], including penicillins and cephalosporins, have profoundly impacted global health, and they continue to inspire the design of novel lactam-based therapeutics. In recent years, attention has shifted toward medium-sized ring and large-sized ring lactams. Seven-membered lactams have been reported to exhibit promising activity profiles and unique mechanisms of action [24]. For example, diltiazem and clentiazem are FDA-approved drugs that exhibit bioactivity in the central nervous system, in addition to other therapeutic actions [25,26]. Cilazapril and benazepril are angiotensin-converting enzyme inhibitors used for the treatment of hypertension and congestive heart failure [27] (Figure 1). The seven-membered derivatives of the phytochemical piperlongumin (another lactam) demonstrate multimodal anticancer activity, targeting various cancer-associated pathways while demonstrating lower toxic to normal cells [28,29]. From the above considerations, it is reasonable to postulate that modification of isolongifolenone with a lactam moiety may increase its anticancer activity.

**Figure 2:**
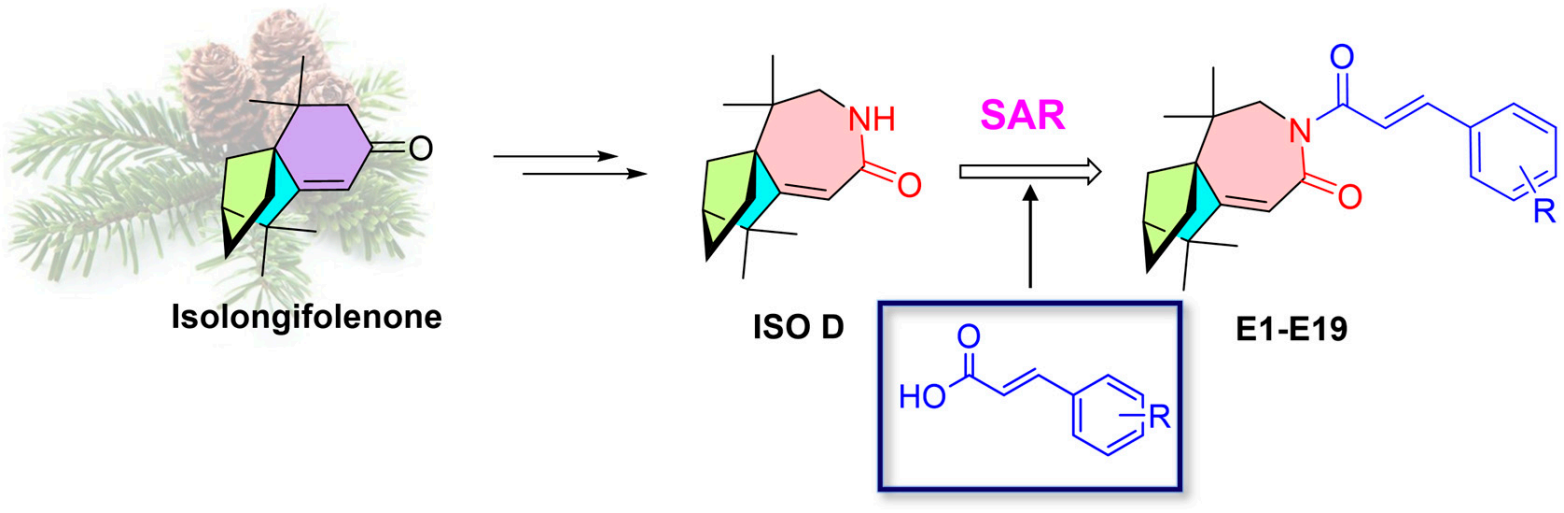
Design strategy for the target compounds (**E1**–**E19**).

Hence, we designed a series of isolongifolenone-based lactam analogues, through introducing a lactam ring and a cinnamoyl moiety, to find derivatives with greater antitumor activity (Figure 2). In the present study, we describe the synthesis of this novel series of isolongifolenone analogues and their enhanced anticancer potency against several cancer cell lines in vitro and in vivo. All derivatives were characterized by ^1^H NMR, ^13^C NMR, and HRMS analysis. Their anti-proliferative activities were then screened in vitro against three human tumor cell lines (MCF-7, A549, and HepG2) and one normal cell line (LO2). The structure-activity relationship for these analogues was then considered. Next, the principal anti-tumor mechanism of derivative **E10** was investigated through fluorescence staining, flow cytometric analysis, and Western blotting. Furthermore, the anti-tumor efficacy of **E10** was comprehensively evaluated using a three-dimensional (3D) cell culture system and a zebrafish xenograft tumor model. Together, the results of this study provide evidence of the potential value of derivative **E10** in the treatment of breast cancer.

## 2. Results and Discussion

### 2.1. Design and Synthesis

**Scheme 1:**
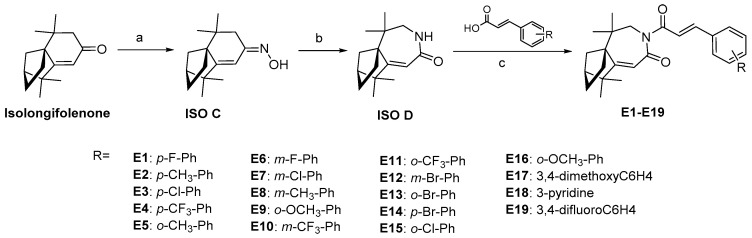
The synthesis route of isolongifolenone derivatives. Reagents and reaction conditions: (a) NaOAc, EtOH, reflux; (b) SOCl_2_, reflux; (c) oxalyl chloride, DCM, 0–5 °C to room temperature.

Isolongifolenone features a rigid fused-ring system and an α,β-unsaturated ketone moiety, which together contribute to its conformational stability and reactivity. The strained tricyclic framework offers a unique three-dimensional architecture that can serve as a versatile scaffold for structural modification, while the enone functional group provides a reactive site for nucleophilic addition, condensation, and other derivatization reactions. Inspired by the natural product bibaxianan and its active component piperlongumine (PL), we designed a series of isolongifolenyl caprolactam derivatives by incorporating a lactam moiety and further functionalizing it with cinnamoyl units. The introduction of the caprolactam ring was expected to improve molecular rigidity and potential bioactivity, while cinnamoyl substitution could modulate electronic and steric properties, potentially enhancing anticancer potency. The synthetic route for preparation of isolongifolenyl caprolactam derivatives is outlined in Scheme 1. Initially, the intermediate compound **ISO C** was synthesized from condensation of isolongifolenone with hydroxylamine hydrochloride, followed by Beckmann rearrangement reaction with thionyl chloride to give the key intermediate **ISO D**. Subsequently, compound **2** was amidated with different cinnamoyl chloride derivatives to afford a series of isolongifolenyl caprolactam derivatives (**E1**–**E19**). The structures of these novel derivatives were subsequently confirmed via ^1^H NMR, ^13^C NMR, and HR-MS (Supplementary Data).

### 2.2. In Vitro Antiproliferative Activity of Isolongifolenone Bicyclocaprolactam Derivatives and Structure-Activity Relationship Studies

After synthesis and characterization, the isolongifolenone bicyclocaprolactam derivatives were evaluated for their in vitro antiproliferative activity against three cancer cell lines (MCF-7, HepG2, A549) using a CCK-8 assay. The resulting IC_50_ values are listed in Table 1. In comparison to Piperlongumine (**PL**), most of the synthesized compounds exhibited strong antiproliferative activity. In general, the antiproliferative activities of most of these compounds were similar, irrespective of whether electron-withdrawing or electron-donating substituents were introduced in the phenoxy ring. However, compound **E10** (which contained an *m*-trifluoromethyl group) exhibited the highest activity, yielding IC_50_ values of 0.32–3.78 µM for the three cancer cell lines tested.

The inclusion of a strong electron-withdrawing group (CF_3_) in the meta position of the phenyl group afforded excellent antiproliferative activity against MCF-7 cancer cells (IC_50_, 0.32 µM). However, the positioning of substituents in the phenyl ring affected the antiproliferative activity of compounds. For example, compounds **E6**–**E8**, **E10**, and **E12**, with a meta-substitution (*m*-F, *m*-Cl, *m*-CH_3_, *m*-Br, and *m*-CF_3_), exhibited increased antiproliferative activity against MCF-7 cells compared with the corresponding para/ortho-substituted analogs. Notably, compounds with a strong electron-withdrawing group on the meta-position (*m*-CF3) exhibited strong antiproliferative activity against all three cancer cell lines, and especially against MCF-7 cells. However, when the phenyl ring was substituted with a pyridine ring (compound **E18**), antiproliferative activity was entirely lost. In addition, compounds with 3,4-disubstitution in the phenyl ring (**E17** and **E19**) exhibited good anticancer activity, indicating that anticancer activity was fairly independent of donor substituents or acceptor substituents in the meta and para positions. In summary, the structure-activity relationships of synthesized isolongifolenone bicyclocaprolactam derivatives indicated that a phenyl ring was necessary for antiproliferative activity and that a meta-substitution on the phenyl ring could enhance this activity.

### 2.3. ***E10*** Induced Cell Apoptosis and Cell Cycle Arrest

**Figure 3:**
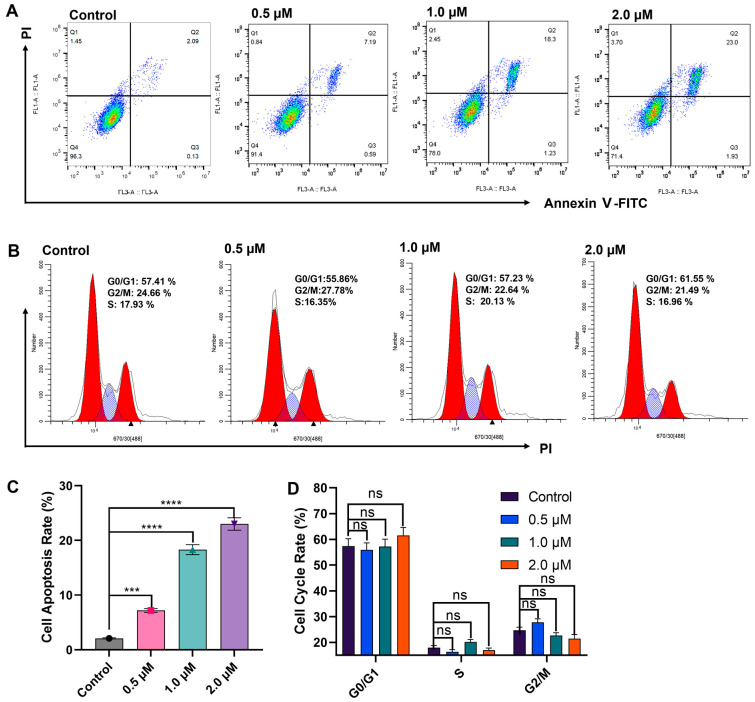
(**A**) Flow cytometry analysis of apoptosis induction in MCF-7 cells treated with compound **E10** (0.5, 1.0, 2.0 µM) for 24 h. The cells were stained with Annexin V-FITC/PI and analyzed by flow cytometry; Q1—Necrotic cells, Q2—Late apoptotic cells, Q3—Early apoptotic cells, Q4—Viable cells. (**B**) Flow cytometry analysis of cell cycle in MCF-7 cells treated with compound **E10** (0.5, 1.0, 2.0 µM) for 24 h. The cells were stained with PI and then analyzed by flow cytometry. (**C**) Quantitative comparison of apoptotic phases in MCF-7 cells. (**D**) Quantitative comparison of cell cycle phase. All assays were performed in 3 independent experiments. Data are shown as mean ± SD. Statistical analysis was performed using one-way ANOVA followed by Dunnett’s post hoc test versus the control group. Significance is indicated as ns (not significant), *** *p* < 0.001, **** *p* < 0.0001.

The ability of drugs to induce cancer cell apoptosis is a key indicator of their chemotherapeutic potential. As noted above, compound **E10** demonstrated significant anticancer activity against the human breast cancer cell line MCF-7 (IC_50_, 0.32 ± 0.47 μM), indicating strong cytotoxic potential. Based on these results, **E10** was selected for further mechanistic evaluation to assess its pro-apoptotic effects. To examine the apoptotic effects of **E10**, an Annexin V-FITC dual-staining assay (with flow cytometric analysis) was performed. As shown in Figure 3A (also Figure 3C), treatment with increasing concentrations of **E10** induced a marked elevation in apoptotic cell populations. The observed apoptosis rates were 7.78%, 19.53%, and 24.93% for the respective concentrations tested, demonstrating a clear dose-dependent increase in apoptosis.

As a cell progresses into different cell cycle phases, its DNA content varies because of DNA synthesis (which is required to prepare additional chromosome copies prior to cell division). Consequently, a flow cytometric analysis of DNA content can provide information on the number of cells in different cell cycle phases. As shown in Figure 3B,D), compound **E10** induced a concentration-dependent increase in the number of cells in the G0/G1 phase and decreased the number of cells in the G2/M phase. These findings suggest that **E10** induces cell cycle arrest at the G0/G1 phase.

### 2.4. DNA Damage Studies of Compound ***E10***

**Figure 4:**
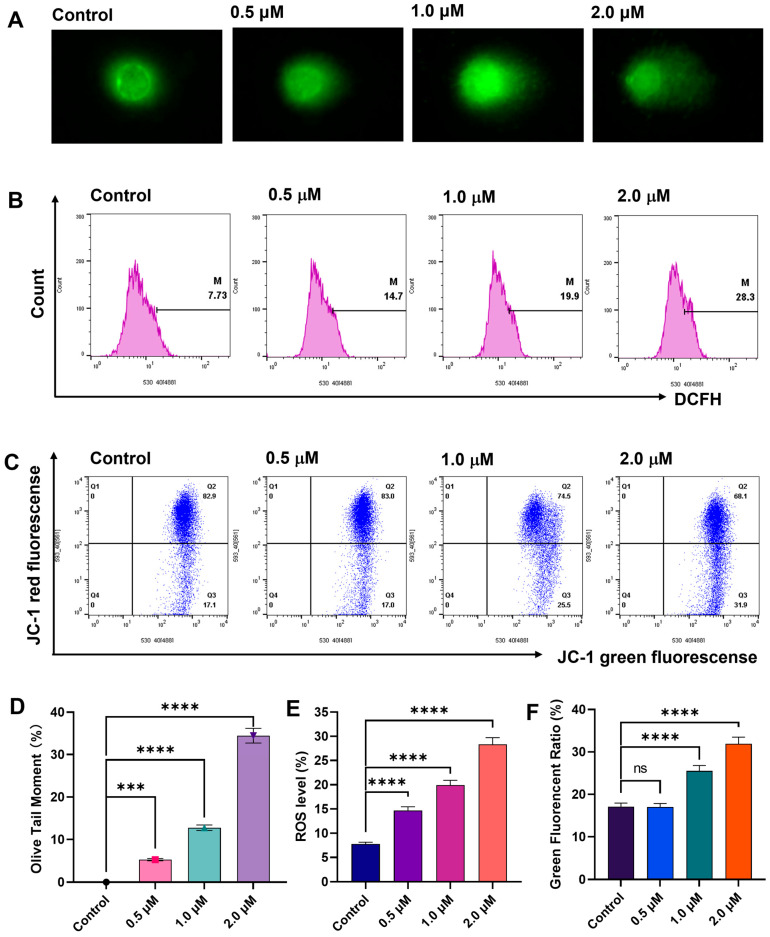
(**A**) Influence of **E10** treatment on MCF-7 COMET formation. (**B**) Flow cytometry analysis of intracellular ROS in MCF-7 cells treated with compound **E10** (0.5, 1.0, 2.0 µM) for 24 h. DCFH-DA was used as a ROS indicator. (**C**) Flow cytometry analysis of intracellular ROS in MCF-7 cells treated with compound **E10** (0.5, 1.0, 2.0 µM) for 24 h. JC-1 was used as a probe for MMP; Q2–Cells with normal ΔΨm, Q3–Cells with depolarized mitochondria (loss of ΔΨm). (**D**) Relative quantification of comet tail moment. (**E**) Relative quantification of ROS. (**F**) Relative quantification of MMP damage. All assays were performed in 3 independent experiments. Data are shown as mean ± SD. Statistical analysis was performed using one-way ANOVA followed by Dunnett’s post hoc test versus the control group. Significance is indicated as ns (not significant), *** *p* < 0.001, **** *p* < 0.0001.

DNA fragmentation is a hallmark of apoptosis, mitotic catastrophe, or both [30]. DNA damage in MCF-7 cells was investigated by single-cell gel electrophoresis in an agarose gel matrix (the ‘comet assay’). In this assay, the length of the ‘comet tail’ observed after EB staining of the cell nuclei is an indicator of the extent of DNA damage. As shown in the control group (Figure 4A,D), DNA from MCF-7 cells does not appear comet-like after EB staining. However, after treatment with compound **E10** for 24 h, the DNA from MCF-7 cells demonstrated well-formed comets after EB staining, and the difference was statistically significant (when compared with untreated cells). These results indicate that **E10** induced DNA fragmentation and provide further evidence of **E10**-induced apoptosis.

### 2.5. ***E10*** Induced ROS Generation and Loss of MMP

Oxidative stress is known to increase intracellular reactive oxygen species (ROS) levels and to contribute to cell apoptosis and autophagy [31]. To investigate whether **E10** induced intracellular ROS accumulation, ROS levels in MCF-7 cells were determined by flow cytometric assay using DCFH-DA as a redox-sensitive probe. As shown in Figure 4B,E, after 24 h treatment with **E10**, intracellular ROS levels were markedly increased (compared to the control group). These results demonstrate that **E10** increased ROS generation in MCF-7 cells.

If intracellular ROS levels rise above a certain threshold, the mitochondria may become damaged [32]. After confirming that **E10** treatment caused a rapid accumulation of intracellular ROS, mitochondrial function in MCF-7 cells after **E10** treatment was next evaluated. In these experiments, MCF-7 cells were incubated with **E10** for 24 h, and mitochondrial membrane potential (MMP) was then evaluated by flow cytometry using JC-1 as an indicator. While red fluorescence decreased markedly after **E10** treatment (Figure 4C,F), green fluorescence was observed to increase in a concentration-dependent manner. These changes in the red/green fluorescence ratio are indicative of mitochondrial depolarization, a decrease in mitochondrial function, and potentially damage to mitochondria. Hence, **E10** treatment induced a rapid, concentration-dependent reduction in MMP and a decrease in mitochondrial function.

### 2.6. ***E10*** Induced Autophagy in MCF-7 Cells

Autophagy is a cellular process that involves the breakdown and recycling of damaged cellular components, and an accumulation of autophagic vacuoles is a hallmark of this process. The cytoplasmic microtubule-associated protein light chain 3-I (LC3-I) is converted into LC3-II, and this specifically associates with autophagosome membranes. The ratio of LC3-II to LC3-I (LC3-II/LC3-I) thus serves as a marker for autophagic activity [33]. In the early stages of autophagosome formation, the adaptor protein p62 binds to LC3 and is subsequently incorporated into the autophagosome membrane, ultimately undergoing degradation in lysosomes. Therefore, the expression level of p62 is commonly used as an indicator of autophagic flux, and an accumulation or reduction of p62 is an indicator of whether autophagy is impaired or proceeding normally [34]. Mammalian target of rapamycin (mTOR), a central regulator of cell growth, proliferation, and apoptosis, also serves as a key negative regulator of autophagy [35].

**Figure 5:**
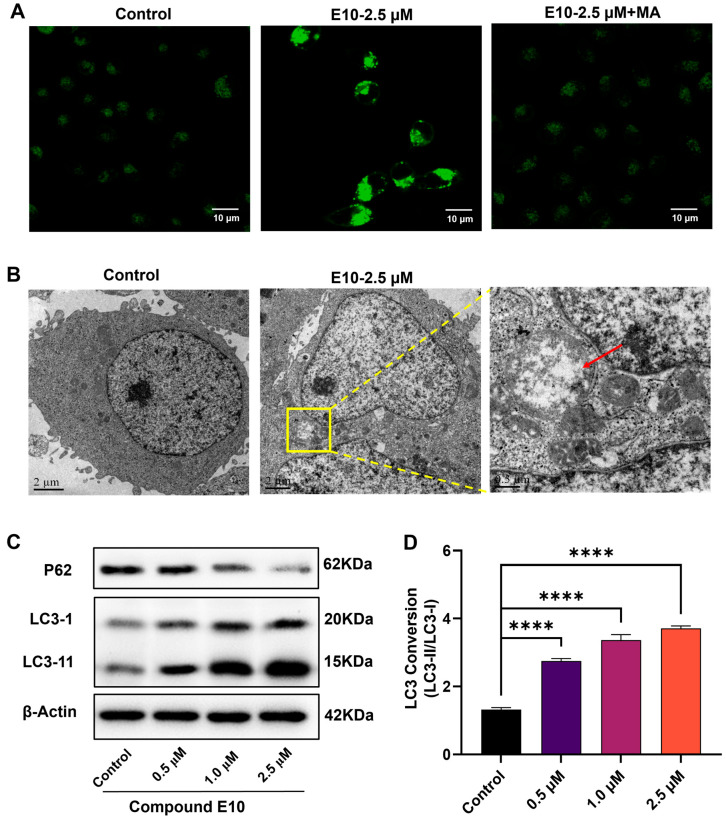
(**A**) MDC staining demonstrating autophagy induction by **E10** in MCF-7 cells. (**B**) Representative TEM images of **E10**-treated MCF-7 cells and untreated MCF-7 cells. The boxed area in the E10 panel is shown at higher magnification on the right; the red arrow marks an autophagosome (double membrane). (**C**) Effect of **E10** on autophagic marker proteins (p62, LC3-I, and LC3-II) in MCF-7 cells. (**D**) Relative densitometric ratios of LC3-II/LC3-I. LC3 conversion (LC3-II/LC3-I) quantified from blots in (**C**). Values are mean ± SD, *n* = 3 independent experiments. Statistical analysis was performed using one-way ANOVA followed by Dunnett’s post hoc test versus the control group. Significance is indicated as **** *p* < 0.0001.

To investigate whether **E10** induced autophagy in MCF-7 cells, we performed Monodansylcadaverine (MDC) staining, transmission electron microscopy (TEM), and Western blot analysis to evaluate autophagy-related markers. MDC is a fluorescent probe commonly used to detect the formation of autophagic vacuoles, as it specifically labels autophagosomes [36]. As shown in Figure 5A, **E10** treatment significantly increased the number of punctate green fluorescence signals (compared with the control group), indicating enhanced autophagosome formation. As a control, cells that were co-treated with 3-methyladenine (3-MA), a pharmacological inhibitor of autophagosome formation through PI3K blockade, exhibited a marked reduction in fluorescence intensity [37]. Additionally, TEM analysis revealed the presence of membrane-bound autophagic vesicles in **E10**-treated MCF-7 cells, providing ultrastructural evidence that **E10** promotes autophagy in MCF-7 cells (Figure 5B).

LC-3 can be considered a core protein of autophagy. In agreement with the above results, Western blot analyses indicated that LC3-I and LC3-II expression levels were up-regulated after **E10** treatment. Moreover, the ratio of LC3-II/LC3-I increased gradually when MCF-7 cells were treated with increasing concentrations of **E10** (Figure 5C,D). Together with the observed decrease in p62. The above results provide evidence that compound **E10** induced autophagy in MCF-7 cells.

### 2.7. ***E10*** Induced Cell Death via the AMPK-mTOR Pathway

**Figure 6:**
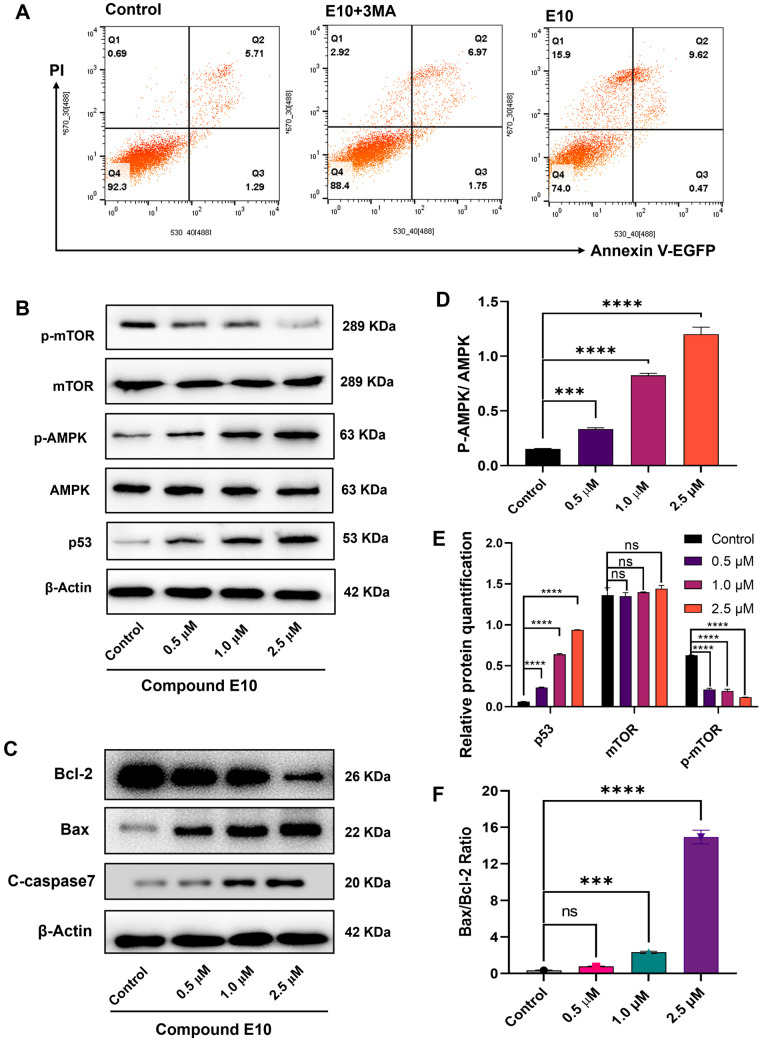
(**A**) Flow cytometry analysis of apoptosis induction in MCF-7 cells treated with compound **E10** or **E10** + 3MA for 24 h. Q1—Necrotic cells, Q2—Late apoptotic cells, Q3—Early apoptotic cells, Q4—Viable cells. (**B**) Effect of **E10** on proteins in the AMPK-mTOR pathway (p-mTOR, mTOR, pAMPK, AMPK, p53) of MCF-7 cells. (**C**) Effect of **E10** on the apoptosis-associated proteins Bcl-2, Bax, and cleaved caspase-7 (C-Caspase3) in MCF-7 cells. (**D**) pAMPK/AMPK ratio. p-AMPK normalized to total AMPK; values relative to vehicle. Mean ± SD (*n* = 3). (**E**) Relative protein quantification of p53, mTOR, and p-mTOR. p53 and total mTOR normalized to β-actin; p-mTOR normalized to total mTOR; each expressed relative to Control. Mean ± SD (*n* = 3). (**F**) Relative protein ratio of Bax and Bcl-2. Bax and Bcl-2 each normalized to β-actin; Bax/Bcl-2 computed per lane and expressed relative to vehicle. Mean ± SD (*n* = 3). Statistical analysis was performed using one-way ANOVA followed by Dunnett’s post hoc test versus the control group. Significance is indicated as ns (not significant), *** *p* < 0.001, **** *p* < 0.0001.

Autophagy has bidirectional regulation effects on cell survival [38]. To confirm the role of autophagy in **E10**-induced cell death, 3-MA was used as an autophagy blocker. As shown in Figure 6A, while cell apoptosis was significantly increased in **E10**-treated MCF-7 cells (in comparison with the control group), the cell apoptosis rate was lower after co-treatment with **E10** and 3-MA. Hence, 3-MA reversed **E10**-induced apoptosis, and autophagy was a requirement for **E10**-induced cell apoptosis.

The AMPK/mTOR signaling pathway is a key regulator of autophagy and is frequently activated in cancer cells [39,40]. To investigate the role of this pathway in **E10**-induced autophagy, AMPK and mTOR expression levels were examined in **E10**-treated MCF-7 cells. As shown in Figure 6B–E, **E10** treatment strongly decreased mTOR phosphorylation levels and increased AMPK phosphorylation levels, which is consistent with the proposal that **E10** induced autophagy via the AMPK-mTOR pathway.

Autophagy is regulated by an intricate network of signaling cascades. P53 has been shown to regulate both AMPK activation and mTOR inhibition [41,42]. P53 also operates at mitochondria to promote cell death [43]. To further investigate the role of P53, the expression levels of apoptosis-associated proteins (Cleaved Caspase-7, Bax, Bcl-2) and p53 were measured using Western blot. As shown in Figure 6B,C, **E10**-treatment induced higher levels of the pro-apoptotic protein Bax (compared with the control group). Conversely, the expression level of anti-apoptotic protein Bcl-2 was down-regulated in the **E10**-treatment group. Furthermore, the cleaved caspase-7 and p53 expression levels were dramatically up-regulated by compound **E10** in a dose-dependent manner. These results provide further evidence that the apoptotic effects of **E10** were exerted via the mitochondrial pathway.

### 2.8. Antiproliferative Activity of ***E10*** in 3D Spheroid Tumor Model

**Figure 7:**
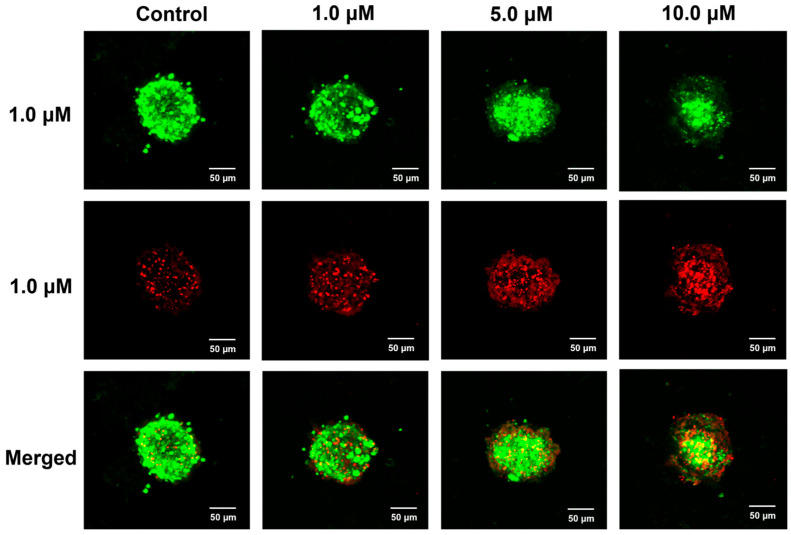
Representative images of MCF-7 cells after treatment with **E10** (1.0, 5.0, 10.0 μM) or Control (0.1% DMSO), stained with Calcein-AM (green, live) and propidium iodide (PI) (red, dead). Images acquired on laser confocal microscopy, filters: green channel 488/525 nm and red channel 535/617 nm. Scale bar = 50 μm.

Three-dimensional (3D) cell culture provides an environment that allows cells to grow and interact with their surroundings in all spatial dimensions, closely mimicking the physiological conditions found in vivo [44]. Hence, the use of 3D cell culture models enhances the accuracy and translational relevance of cellular biology research. To evaluate the long-term antitumor effects of **E10** in a more physiologically relevant model, a 3D spheroid tumor model was established using MCF-7 breast cancer cells. Each spheroid was formed by approximately 2000 MCF-7 cells and cultured for 3 days to allow spheroid maturation. These spheroids were subsequently treated with varying concentrations of **E10** (1, 5, and 10 μM) at 48-h intervals over a period of 4 days. For visualization, the multicellular tumor spheroids (MCTSs) were stained with calcein AM and propidium iodide (PI) in culture medium for 1.5 h. The live/dead cell distribution within the spheroids was then visualized using laser scanning confocal microscopy. As shown in Figure 7, control spheroids primarily exhibited green fluorescence from calcein AM, indicating viable cells, with a minor presence of red fluorescence from PI localized to the spheroid core. This limited cell death is likely attributable to hypoxic and nutrient-deprived conditions resulting from excessive cell proliferation. In contrast, **E10**-treated spheroids exhibited a marked increase in red fluorescence corresponding to necrotic cores, and this became more pronounced with increasing **E10** concentrations. These results indicate that **E10** exerts a dose-dependent inhibitory effect on the viability and structural integrity of MCF-7 MCTSs. Thus, **E10** effectively inhibited the growth of MCF-7 cells in a 3D tumor spheroid model, demonstrating the strong antitumor potential of **E10** under conditions that mimic the in vivo tumor microenvironment.

### 2.9. Compound ***E10*** Suppresses Tumor Growth In Vivo

**Figure 8:**
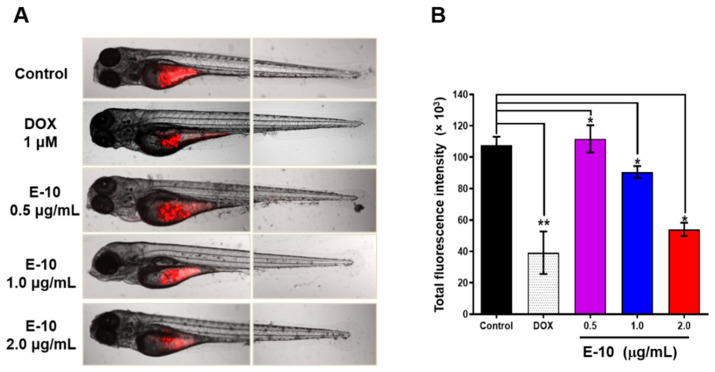
Effect of compound **E10** on MCF-7 breast cancer cells in the xenograft zebrafish model. (**A**) Dil-labeled MCF-7 cells (red) were microinjected into zebrafish embryos and treatment with **E10** (0.5, 1.0 and 2.0 μg/mL) and Dox for 48 h. After 48 h, the proliferationof the xenografts of MCF-7 cells were imaged under a fluorescence microscope. Images were acquired using a 5 × objective lens, with a scale bar representing 100 µm. (**B**) Quantification of the fluorescent area of the tumor xenografts, representing total MCF-7 cells in zebrafish (n = 10/group). All data were presented as the means ± SD. Statistical analysis was performed using one-way ANOVA followed by Dunnett’s post hoc test versus the control group. Significance is indicated as ns (not significant), * *p* < 0.05, ** *p* < 0.01.

Zebrafish have become a well-established model for both anticancer drug discovery and toxicity evaluation. This animal model affords several unique advantages, including rapid development, optical transparency, and genetic conservation with humans [45,46]. These features make zebrafish particularly suitable for high-throughput in vivo screening and mechanistic studies in oncology research. Tumor burden in zebrafish xenografts is commonly quantified from Dil/DiI-labeled fluorescence area or integrated intensity, which correlates with cell number and treatment response [47,48]. To assess the in vivo antitumor efficacy of compound **E10**, we established a zebrafish xenograft model. Briefly, CM-Dil-labeled MCF-7 cells (showing red fluorescence) were microinjected into the yolk sacs of zebrafish embryos. The xenografted zebrafish were then randomly assigned into five groups: three **E10**-treatment groups (0.5 µg/mL, 1.0 µg/mL, and 2.0 µg/mL), a Doxorubicin (Dox) positive control group, and a PBS negative control group. As shown in Figure 8, zebrafish xenografts treated with Dox or **E10** exhibited a notable reduction in fluorescence intensity (when compared with the PBS control group). Notably, treatment with 2.0 µg/mL **E10** induced a significant decrease in fluorescence, corresponding to an inhibition rate of 50.0%, suggesting that **E10** effectively suppressed MCF-7 cell proliferation in vivo. The evidence that **E10** exhibits significant in vivo antitumor activity, along with evidence of its in vitro potency, demonstrates its promise as a potential anticancer candidate.

## 3. Conclusions

A series of new isolongifolenone-based caprolactam derivatives bearing a cinnamoyl unit for use as anticancer agents were designed and synthesized. These synthesized compounds exerted potent inhibition against three cancer cell lines. Among these derivatives, compound **E10** exhibited excellent inhibitory activity against MCF-7 breast cancer cells (IC_50_, 0.32 µM), as well as potent antitumor effects in a three-dimensional (3D) cell culture model. A structure-activity relationship analysis of these results indicated that compounds containing a strong electron-withdrawing group (CF_3_) in the *meta*-position of the phenyl group exhibited enhanced activity. Additional mechanistic studies revealed that **E10** induced cell autophagy by upregulating p53 expression, activating AMPK, and inhibiting mTOR phosphorylation. Furthermore, **E10** induced apoptosis by regulating the expression of apoptosis-associated proteins. Importantly, **E10** effectively inhibited the proliferation of MCF-7 cells in a zebrafish xenograft model at low concentrations. Together, these results provide firm evidence that an isolongifolenone-based caprolactam derivative (**E10**) could be developed as a potential cancer treatment agent.

## 4. Materials and Methods

### 4.1. Chemistry

All chemical reagents were purchased from commercial suppliers (Adamas, Shanghai, China; Meryer, Shanghai, China; Bidepharm, Shanghai, China). All purchased reagents were used directly without further purification. Thin layer chromatography (TLC, UV 254 nm) on a silica gel plate (0.25 mm, Qingdao Ocean Chemical Ltd., Qingdao, China) was used to verify the subsequent responses. Nuclear magnetic resonance (NMR) spectroscopy was recorded at room temperature on a Bruker AVANCE 600/400 instrument in CDCl_3_ or DMSO-*d6* solutions with tetramethylsilane (TMS) as an internal standard. The following abbreviations are used: s (singlet), br (broad signal), d (doublet), dd (doublet of doublets), t (triplet), q (quartet), and m (multiplet). Coupling constants were reported in Hz. High-resolution mass spectra were recorded on MicroQTOF II.

#### 4.1.1. General Procedure for the Synthesis of Compound **ISO C**

A three-necked flask was charged with isolongifolenone (1.30 g, 6 mmol), hydroxylamine hydrochloride (0.42 g, 6.1 mmol), and 50 mL of ethyl alcohol, with sodium acetate anhydrous (6 mmol) as catalyst. The mixture was stirred and refluxed at 78 °C for 4 h. After the reaction, the solvent was evaporated, and the residue was washed three times with a dilute hydrochloric acid solution. Next, the organic layer was concentrated under reduced pressure (in vacuo). The crude product was then purified by recrystallization from methanol (MeOH), yielding pure compound **ISO C**.

#### 4.1.2. General Procedure for the Synthesis of Compound **ISO D**

Compound **ISO C** (1.40 g, 6 mmol) was dissolved in thionyl chloride (15 mL), and the mixture was stirred at 70 °C for 6 h. After reaction, the rest of the thionyl chloride was removed using a rotary vacuum concentrator under reduced pressure. The residue was dissolved in ethyl acetate and washed with sodium bicarbonate aqueous solution. The organic layer was concentrated *in vacuo*, and the pure compound **ISO D** was obtained from recrystallization in ethyl acetate.

#### 4.1.3. General Procedure for the Synthesis of Derivatives **E1**–**E19**

A cinnamic acid derivative (1 mmol) was dissolved in 10 mL of dichloromethane (DCM), and oxalyl chloride was added at 0 °C. The mixture was then stirred at room temperature for 30 min. Afterward, the solvent was removed. The residue was dissolved in fresh DCM, and this solution was added to a DCM solution of compound **ISO B** (10 mL), with triethylamine (TEA) as the catalyst. The mixture was stirred at room temperature for 6 h. After the reaction, the solvent was removed under reduced pressure. The pure targeted compounds (**E1**–**E19**) were then isolated by silica gel column chromatography.

*5,5,9,9-Tetramethyl-3-(3-(4-fluorophenyl)acryloyl)-4,5,6,7,8,9-hexahydro-5a,8-methanobenzo[d]azepin-2(3H)-one (***E1***).* White solid, yield 53%. mp. 128.5—129.3 °C; ^1^H NMR (600 MHz, CDCl_3_) δ: 7.64 (d, *J* = 15.5 Hz, 1H), 7.54 (dd, *J* = 8.6, 5.5 Hz, 2H), 7.21 (d, *J* = 15.6 Hz, 1H), 7.04 (t, *J* = 8.6 Hz, 2H), 5.74 (s, 1H), 4.11 (d, *J* = 14.4 Hz, 1H), 3.56 (d, *J* = 14.4 Hz, 1H), 1.97 (s, 1H), 1.89–1.91 (m, 1H), 1.78–1.83 (m, 1H), 1.72 (d, *J* = 7.9 Hz, 1H), 1.53–1.57 (m, 1H), 1.40 (d, *J* = 10.1 Hz, 1H), 1.28–1.38 (m, 1H), 1.17 (s, 3H), 1.09 (s, 3H), 1.06 (s, 3H), 0.96 (s, 3H). ^13^C NMR (150 MHz, CDCl_3_) δ: 176.74, 170.04, 169.81, 141.33, 130.07, 130.01, 121.86, 115.87, 115.73, 114.14, 63.74, 49.13, 46.07, 45.52, 37.68, 34.32, 28.88, 28.79, 25.37, 24.81, 24.21, 23.09. HRMS (ESI) *m*/*z*: 381.2104 [M + H^+^], calcd. for C_24_H_28_FNO_2_ 382.2184.

*5,5,9,9-Tetramethyl-3-(3-(4-tolyl)acryloyl)-4,5,6,7,8,9-hexahydro-5a,8-methanobenzo[d]azepin-2(3H)-one (***E2***).* White solid, yield 50%. mp. 156.1—156.7 °C; ^1^H NMR (600 MHz, CDCl_3_) δ: 7.68 (d, *J* = 15.5 Hz, 1H), 7.45 (d, *J* = 8.1 Hz, 2H), 7.25 (d, *J* = 15.5 Hz, 1H), 7.16 (d, *J* = 7.9 Hz, 2H), 5.74 (s, 1H), 4.10 (d, *J* = 14.5 Hz, 1H), 3.56 (d, *J* = 14.5 Hz, 1H), 2.35 (s, 3H), 1.96 (s, 1H), 1.88–1.93 (m, 1H), 1.77–1.82 (m, 1H), 1.71 (d, *J* = 10.2, 1H), 1.51–1.57 (m, 1H), 1.39 (d, *J* = 10.1 Hz, 1H), 1.27–1.32 (m, 1H), 1.16 (s, 3H), 1.09 (s, 3H), 1.05 (s, 3H), 0.96 (s, 3H). ^13^C NMR (150 MHz, CDCl_3_) δ: 176.42, 170.11, 170.02, 142.90, 140.15, 132.53, 129.44, 128.25, 120.99, 114.25, 63.70, 49.14, 46.07, 45.47, 37.68, 34.34, 28.90, 28.76, 25.37, 24.82, 24.21, 23.12, 21.46. HRMS (ESI) *m*/*z*: 377.2355 [M + H^+^], calcd. for C_25_H_31_NO_2_ 378.2397.

*5,5,9,9-Tetramethyl-3-(3-(4-chlorophenyl)acryloyl)-4,5,6,7,8,9-hexahydro-5a,8-methanobenzo[d]azepin-2(3H)-one (***E3***).* White solid, yield 47%. mp. 195.3—195.9 °C; ^1^H NMR (600 MHz, CDCl_3_) δ: 7.64 (d, *J* = 15.6 Hz, 1H), 7.51 (d, *J* = 8.5 Hz, 2H), 7.34 (d, *J* = 8.5 Hz, 2H), 7.27 (d, *J* = 15.7 Hz, 1H), 5.76 (s, 1H), 4.14 (d, *J* = 14.5 Hz, 1H), 3.58 (d, *J* = 14.4 Hz, 1H), 1.98 (m, 1H), 1.91–1.95 (m, 1H), 1.80–1.84 (m, 1H), 1.74 (d, *J* = 10.1, 1H), 1.60–1.53 (m, 1H), 1.43 (d, *J* = 10.2, 1H), 1.30–1.35 (m, 1H), 1.19 (s, 3H), 1.12 (s, 3H), 1.08 (s, 3H), 0.98 (s, 3H). ^13^C NMR (150 MHz, CDCl_3_) δ: 176.86, 170.04, 169.71, 141.04, 135.58, 133.81, 129.37, 128.96, 122.68, 114.08, 63.75, 49.12, 46.06, 45.54, 37.67, 34.31, 28.87, 28.79, 25.36, 24.81, 24.22, 23.08. HRMS (ESI) *m*/*z*: 397.180 [M + H^+^], calcd. for C_24_H_28_ClNO_2_ 396.2537.

*5,5,9,9-Tetramethyl-3-(3-(4-(trifluoromethyl)phenyl)acryloyl)-4,5,6,7,8,9-hexahydro-5a,8-methanobenzo[d]azepin-2(3H)-one (***E4***)*. White solid, yield 55%. mp. 170.6—171.0 °C; ^1^H NMR (600 MHz, CDCl_3_) δ: 7.66 (d, *J* = 2.8 Hz, 1H), 7.64 (d, *J* = 4.6 Hz, 2H), 7.60 (d, *J* = 8.3 Hz, 2H), 7.33 (d, *J* = 15.6 Hz, 1H), 5.75 (s, 1H), 4.13 (d, *J* = 14.4 Hz, 1H), 3.56 (d, *J* = 14.4 Hz, 1H), 1.97 (s, 1H), 1.89–1.94 (m, 1H), 1.89–1.94 (m, 1H), 1.72 (d, *J* = 10.2, 1H), 1.53–1.58 (m, 1H), 1.41 (d, *J* = 10.2 Hz, 1H), 1.28–1.33 (m, 1H), 1.17 (s, 3H), 1.10 (s, 3H), 1.06 (s, 3H), 0.96 (s, 3H). ^13^C NMR (150 MHz, CDCl_3_) δ: 177.27, 170.17, 169.60, 140.38, 138.87, 128.40, 125.80, 125.77, 124.79, 114.09, 63.92, 49.25, 46.19, 45.72, 37.81, 34.42, 28.99, 28.94, 25.48, 24.94, 24.35, 23.19. HRMS (ESI) *m*/*z*: 431.2072 [M + H^+^], calcd. for C_25_H_28_F_3_NO_2_ 432.2158.

*5,5,9,9-Tetramethyl-3-(3-(2-tolyl)acryloyl)-4,5,6,7,8,9-hexahydro-5a,8-methanobenzo[d]azepin-2(3H)-one (***E5***).* White solid, yield 55%. mp. 138.3—138.9 °C; ^1^H NMR (600 MHz, CDCl_3_) δ: 7.96 (d, *J* = 15.4 Hz, 1H), 7.62 (d, *J* = 8.3 Hz, 1H), 7.23 (t, *J* = 7.4 Hz, 1H), 7.15–7.21 (m, 3H), 5.75 (s, 1H), 4.11 (d, *J* = 14.4 Hz, 1H), 3.57 (d, *J* = 14.4 Hz, 1H), 2.45 (s, 3H), 1.96 (s, 1H), 1.89–1.91 (m, 1H), 1.78–1.82 (m, 1H), 1.72 (d, *J* = 10.1, 1H), 1.52–1.58 (m, 1H), 1.40 (d, *J* = 10.2 Hz, 1H), 1.28–1.33 (m, 1H), 1.16 (s, 3H), 1.09 (s, 3H), 1.06 (s, 3H), 0.97 (s, 3H). ^13^C NMR (150 MHz, CDCl_3_) δ: 176.51, 170.12, 169.99, 140.12, 137.62, 134.17, 130.61, 129.56, 126.75, 126.17, 123.11, 114.19, 63.72, 49.17, 46.07, 45.48, 37.69, 34.34, 28.89, 28.77, 25.37, 24.82, 24.21, 23.11, 19.84. HRMS (ESI) *m*/*z*: 377.2355 [M + H^+^], calcd. for C_25_H_31_NO_2_ 378.2438.

*5,5,9,9-Tetramethyl-3-(3-(3-fluorophenyl)acryloyl)-4,5,6,7,8,9-hexahydro-5a,8-methanobenzo[d]azepin-2(3H)-one (***E6***).* White solid, yield 58%. mp. 105.7—106.1 °C; ^1^H NMR (600 MHz, CDCl_3_) δ: 7.61 (d, *J* = 15.5 Hz, 1H), 7.30–7.33 (m, 2H), 7.25 (d, *J* = 15.4 Hz, 2H), 7.01–7.06 (m, 1H), 5.74 (s, 1H), 4.12 (d, *J* = 14.4 Hz, 1H), 3.55 (d, *J* = 14.4 Hz, 1H), 1.97 (s, 1H), 1.89–1.94 (m, 1H), 1.78–1.83 (m, 1H), 1.72 (d, *J* = 10.2, 1H), 1.53–1.58 (m, 1H), 1.40 (d, *J* = 10.2 Hz, 1H), 1.28–1.33 (m, 1H), 1.17 (s, 3H), 1.09 (s, 3H), 1.06 (s, 3H), 0.96 (s, 3H). ^13^C NMR (150 MHz, CDCl_3_) δ: 176.92, 170.02, 169.63, 140.96, 140.94, 130.22, 124.19, 124.17, 123.47, 116.65, 116.51, 114.45, 114.30, 114.05, 63.75, 49.11, 46.06, 45.56, 37.66, 34.30, 28.86, 28.80, 25.36, 24.81, 24.22, 23.07. HRMS (ESI) *m*/*z*: 381.2104 [M + H^+^], calcd. for C_24_H_28_FNO_2_ 382.2182.

*5,5,9,9-Tetramethyl-3-(3-(3-chlorophenyl)acryloyl)-4,5,6,7,8,9-hexahydro-5a,8-methanobenzo[d]azepin-2(3H)-one (***E7***).* White solid, yield 40%. mp. 125.1—126.2 °C; ^1^H NMR (600 MHz, CDCl_3_) δ: 7.58 (d, *J* = 15.5 Hz, 1H), 7.55 (s, 1H), 7.41 (dt, *J* = 6.9, 1.7 Hz, 1H), 7.32–7.27 (m, 2H), 7.27 (s, 1H), 5.75 (s, 1H), 4.12 (d, *J* = 14.4 Hz, 1H), 3.55 (d, *J* = 14.4 Hz, 1H), 1.97 (s, 1H), 1.89–1.94 (m, 1H), 1.78–1.83 (m, 1H), 1.72 (d, *J* = 10.1, 1H), 1.53–1.59 (m, 1H), 1.41 (d, *J* = 10.2 Hz, 1H), 1.28–1.33 (m, 1H), 1.17 (s, 3H), 1.10 (s, 3H), 1.06 (s, 3H), 0.96 (s, 3H). ^13^C NMR (150 MHz, CDCl_3_) δ: 176.96, 170.02, 169.58, 140.71, 137.14, 134.74, 129.93, 129.62, 127.70, 126.55, 123.52, 114.04, 63.76, 49.11, 46.06, 45.57, 37.66, 34.30, 28.86, 28.81, 25.36, 24.81, 24.23, 23.06. HRMS (ESI) *m*/*z*: 397.1809 [M + H^+^], calcd. for C_24_H_28_ClNO_2_ 398.1888.

*5,5,9,9-Tetramethyl-3-(3-(4-tolyl)acryloyl)-4,5,6,7,8,9-hexahydro-5a,8-methanobenzo[d]azepin-2(3H)-one (***E8***).* White solid, yield 58%. mp. 104.6—105.3 °C; ^1^H NMR (600 MHz, CDCl_3_) δ: 7.66 (d, *J* = 15.6 Hz, 1H), 7.39 (s, 1H), 7.34 (d, *J* = 7.9 Hz, 1H), 7.29 (s, 1H), 7.23 (t, *J* = 7.6 Hz, 1H), 7.15 (d, *J* = 7.6 Hz, 1H), 5.75 (s, 1H), 4.11 (d, *J* = 14.5 Hz, 1H), 3.56 (d, *J* = 14.5 Hz, 1H), 2.35 (s, 3H), 1.95 (s, 1H), 1.82–1.93 (m, 1H), 1.77–1.81 (m, 1H), 1.71 (d, *J* = 10.1, 1H), 1.52–1.57 (m, 1H), 1.39 (d, *J* = 10.1 Hz, 1H), 1.27–1.33 (m, 1H), 1.16 (s, 3H), 1.09 (s, 3H), 1.05 (s, 3H), 0.95 (s, 3H). ^13^C NMR (150 MHz, CDCl_3_) δ: 184.68, 176.53, 170.01, 142.95, 138.31, 135.20, 130.68, 128.59, 128.56, 125.74, 121.81, 114.21, 63.71, 49.13, 46.07, 45.48, 37.68, 34.33, 28.89, 28.77, 25.37, 24.81, 24.21, 23.10, 21.31. HRMS (ESI) *m*/*z*: 377.2355 [M + H^+^], calcd. for C_25_H_31_NO_2_ 378.2441.

*5,5,9,9-Tetramethyl-3-(3-(2-methoxyphenyl)acryloyl)-4,5,6,7,8,9-hexahydro-5a,8-methanobenzo[d]azepin-2(3H)-one (***E9***).* White solid, yield 50%. mp. 162.7—163.5 °C; ^1^H NMR (600 MHz, CDCl_3_) δ: 8.08 (d, *J* = 15.7 Hz, 1H), 7.61 (d, *J* = 7.7 Hz, 1H), 7.35 (d, *J* = 15.8 Hz, 1H), 7.32 (d, *J* = 6.9 Hz, 1H), 6.94 (t, *J* = 7.5 Hz, 1H), 6.90 (d, *J* = 8.3 Hz, 1H), 5.76 (s, 1H), 4.12 (d, *J* = 14.4 Hz, 1H), 3.89 (s, 3H), 3.59 (d, *J* = 14.4 Hz, 1H), 1.97 (s, 1H), 1.89–1.94 (m, 1H), 1.79–1.84 (m, 1H), 1.73 (d, *J* = 10.2 Hz, 1H), 1.53–1.59 (m, 1H), 1.41 (d, *J* = 10.1 Hz, 1H), 1.30–1.34 (m, 1H), 1.18 (s, 3H), 1.11 (s, 3H), 1.07 (s, 3H), 0.98 (s, 3H). ^13^C NMR (150 MHz, CDCl_3_) δ: 176.14, 170.34, 169.94, 158.24, 137.93, 131.04, 128.50, 124.35, 122.24, 120.58, 114.31, 111.05, 63.67, 55.53, 49.17, 46.07, 45.41, 37.70, 34.35, 28.92, 28.72, 25.38, 24.82, 24.17, 23.14. HRMS (ESI) *m*/*z*: 393.2304 [M + H^+^], calcd. for C_25_H_31_NO_3_ 394.2385.

*5,5,9,9-Tetramethyl-3-(3-(3-(trifluoromethyl)phenyl)acryloyl)-4,5,6,7,8,9-hexahydro-5a,8-methanobenzo[d]azepin-2(3H)-one (***E10***)*. White solid, yield 43%. mp. 125.4—125.9 °C; ^1^H NMR (600 MHz, CDCl_3_) δ: 7.81 (s, 1H), 7.74 (d, *J* = 8.0 Hz, 1H), 7.68 (d, *J* = 15.6 Hz, 1H), 7.61 (d, *J* = 7.8 Hz, 1H), 7.50 (t, *J* = 7.8 Hz, 1H), 7.34 (d, *J* = 15.6 Hz, 1H), 5.78 (s, 1H), 4.15 (d, *J* = 14.5 Hz, 1H), 3.59 (d, *J* = 14.4 Hz, 1H), 1.99 (s, 1H), 1.92–1.97 (m, 1H), 1.81–1.85 (m, 1H), 1.75 (d, *J* = 10.2, 1H), 1.56–1.61 (m, 1H), 1.43 (d, *J* = 10.2 Hz, 1H), 1.31–1.36 (m, 1H), 1.19 (s, 3H), 1.12 (s, 3H), 1.09 (s, 3H), 0.99 (s, 3H). ^13^C NMR (150 MHz, CDCl_3_) δ: 177.08, 170.02, 169.49, 140.41, 136.11, 131.35, 131.32, 129.22, 126.12, 126.09, 124.59, 124.56, 124.02, 113.99, 63.77, 49.12, 46.06, 45.58, 37.67, 34.30, 28.86, 28.80, 25.35, 24.80, 24.22, 23.07. HRMS (ESI) *m*/*z*: 431.2072 [M + H^+^], calcd. for C_25_H_28_F_3_NO_2_ 432.1982.

*5,5,9,9-Tetramethyl-3-(3-(2-(trifluoromethyl)phenyl)acryloyl)-4,5,6,7,8,9-hexahydro-5a,8-methanobenzo[d]azepin-2(3H)-one (***E11***)*. White solid, yield 60%. mp. 199.7—200.4 °C; ^1^H NMR (600 MHz, CDCl_3_) δ: 8.01 (dd, *J* = 15.4, 2.3 Hz, 1H), 7.83 (d, *J* = 7.8 Hz, 1H), 7.69 (d, *J* = 7.9 Hz, 1H), 7.55 (t, *J* = 7.6 Hz, 1H), 7.45 (t, *J* = 7.6 Hz, 1H), 7.28 (d, *J* = 15.3 Hz, 1H), 5.76 (s, 1H), 4.14 (d, *J* = 14.5 Hz, 1H), 3.59 (d, *J* = 14.5 Hz, 1H), 1.99 (s, 1H), 1.92–1.96 (m, 1H), 1.80–1.86 (m, 1H), 1.74 (d, *J* = 10.2 Hz, 1H), 1.56–1.61 (m, 1H), 1.43 (d, *J* = 10.2 Hz, 1H), 1.30–1.36 (m, 1H), 1.19 (s, 3H), 1.11 (s, 3H), 1.09 (s, 3H), 0.99 (s, 3H), 0.85–0.93 (m, 1H). ^13^C NMR (150 MHz, CDCl_3_) δ: 176.99, 170.01, 169.27, 137.13, 134.25, 131.91, 129.02, 128.87, 128.21, 126.26, 125.99, 125.95, 113.95, 63.79, 49.09, 46.06, 45.53, 37.67, 34.26, 28.87, 28.76, 25.34, 24.80, 24.15, 23.02. HRMS (ESI) *m*/*z*: 431.2072 [M + H^+^], calcd. for C_25_H_28_F_3_NO_2_ 432.2157.

*5,5,9,9-Tetramethyl-3-(3-(3-bromophenyl)acryloyl)-4,5,6,7,8,9-hexahydro-5a,8-methanobenzo[d]azepin-2(3H)-one (***E12***).* White solid, yield 46%. mp. 105.2—105.8 °C; ^1^H NMR (600 MHz, CDCl_3_) δ: 7.58 (d, *J* = 15.5 Hz, 1H), 7.55 (s, 1H), 7.41 (dt, *J* = 6.9, 1.7 Hz, 1H), 7.32–7.27 (m, 2H), 7.27 (s, 1H), 5.75 (s, 1H), 4.12 (d, *J* = 14.4 Hz, 1H), 3.55 (d, *J* = 14.4 Hz, 1H), 1.97 (s, 1H), 1.89–1.94 (m, 1H), 1.78–1.83 (m, 1H), 1.72 (d, *J* = 10.1, 1H), 1.53–1.59 (m, 1H), 1.41 (d, *J* = 10.2 Hz, 1H), 1.28–1.33 (m, 1H), 1.17 (s, 3H), 1.10 (s, 3H), 1.06 (s, 3H), 0.96 (s, 3H). ^13^C NMR (150 MHz, CDCl_3_) δ: 176.96, 170.02, 169.58, 140.71, 137.14, 134.74, 129.93, 129.62, 127.70, 126.55, 123.52, 114.04, 63.76, 49.11, 46.06, 45.57, 37.66, 34.30, 28.86, 28.81, 25.36, 24.81, 24.23, 23.06. HRMS (ESI) *m*/*z*: 441.1303 [M + H^+^], calcd. for C_24_H_28_BrNO_2_ 442.1384.

*5,5,9,9-Tetramethyl-3-(3-(2-bromophenyl)acryloyl)-4,5,6,7,8,9-hexahydro-5a,8-methanobenzo[d]azepin-2(3H)-one (***E13***).* White solid, yield 50%. mp. 130.1—131.0 °C; ^1^H NMR (600 MHz, CDCl_3_) δ: 8.01 (d, *J* = 15.4 Hz, 1H), 7.69 (d, *J* = 7.9 Hz, 1H), 7.58 (d, *J* = 9.2 Hz, 1H), 7.30–7.26 (m, 1H), 7.23 (d, *J* = 15.5 Hz, 1H), 7.18 (t, *J* = 7.7 Hz, 1H), 5.74 (s, 1H), 4.12 (d, *J* = 14.4 Hz, 1H), 3.57 (d, *J* = 14.4 Hz, 1H), 1.98–1.95 (m, 1H), 1.92 (td, *J* = 12.4, 3.3 Hz, 1H), 1.83–1.77 (m, 1H), 1.72 (d, *J* = 10.2 Hz, 1H), 1.56 (tdd, *J* = 12.4, 5.9, 3.9 Hz, 1H), 1.40 (d, *J* = 10.1 Hz, 1H), 1.31 (ddd, *J* = 12.4, 11.2, 5.9 Hz, 1H), 1.09 (s, 3H), 1.06 (s, 3H), 0.97 (s, 3H). ^13^C NMR (150 MHz, CDCl_3_) δ: 176.86, 170.01, 169.47, 140.45, 135.29, 133.25, 130.69, 128.11, 127.53, 125.36, 124.83, 114.03, 63.77, 49.12, 46.06, 45.53, 37.68, 34.29, 28.88, 28.77, 25.36, 24.81, 24.19, 23.07. HRMS (ESI) *m*/*z*: 441.1303 [M + H^+^], calcd. for C_24_H_28_BrNO_2_ 442.1375.

*5,5,9,9-Tetramethyl-3-(3-(4-bromophenyl)acryloyl)-4,5,6,7,8,9-hexahydro-5a,8-methanobenzo[d]azepin-2(3H)-one (***E14***).* White solid, yield 50%. mp. 195.7—196.3 °C; ^1^H NMR (600 MHz, CDCl_3_) δ: 7.61 (d, *J* = 15.5 Hz, 1H), 7.49 (d, *J* = 8.5 Hz, 2H), 7.43 (d, *J* = 8.6 Hz, 2H), 7.28 (d, *J* = 15.5 Hz, 1H), 5.76 (s, 1H), 4.13 (d, *J* = 14.4 Hz, 1H), 3.57 (d, *J* = 14.5 Hz, 1H), 1.98 (s, 1H), 1.90–1.96 (m, 1H), 1.79–1.84 (m, 1H), 1.73 (d, *J* = 10.2 Hz, 1H), 1.54–1.60 (m, 1H), 1.42 (d, *J* = 10.2 Hz, 1H), 1.29–1.36 (m, 1H), 1.18 (s, 3H), 1.11 (s, 3H), 1.07 (s, 3H), 0.97 (s, 3H). ^13^C NMR (151 MHz, CDCl_3_) δ: 176.87, 170.02, 169.68, 141.05, 134.24, 131.91, 129.60, 123.91, 122.80, 114.06, 63.74, 49.11, 46.05, 45.54, 37.67, 34.30, 28.87, 28.78, 25.36, 24.80, 24.21, 23.08. HRMS (ESI) *m*/*z*: 441.1303 [M + H^+^], calcd. for C_24_H_28_BrNO_2_ 442.1387.

*5,5,9,9-Tetramethyl-3-(3-(2-chlorophenyl)acryloyl)-4,5,6,7,8,9-hexahydro-5a,8-methanobenzo[d]azepin-2(3H)-one (***E15***).* White solid, yield 45%. mp. 108.5—109.3 °C; ^1^H NMR (600 MHz, CDCl_3_) δ: 8.08 (d, *J* = 15.5 Hz, 1H), 7.72 (d, *J* = 7.4 Hz, 1H), 7.41 (d, *J* = 7.6 Hz, 1H), 7.31–7.24 (m, 4H), 5.76 (s, 1H), 4.14 (d, *J* = 14.4 Hz, 1H), 3.59 (d, *J* = 14.4 Hz, 1H), 1.99 (s, 1H), 1.94 (td, *J* = 12.4, 3.3 Hz, 1H), 1.87–1.79 (m, 1H), 1.74 (d, *J* = 10.2 Hz, 1H), 1.58 (tdd, *J* = 12.4, 5.9, 3.9 Hz, 1H), 1.43 (d, *J* = 10.2 Hz, 1H), 1.36–1.29 (m, 1H), 1.19 (s, 3H), 1.11 (s, 3H), 1.08 (s, 3H), 0.99 (s, 3H). ^13^C NMR (151 MHz, CDCl_3_) δ: 176.84, 170.01, 169.55, 137.92, 134.92, 133.53, 130.51, 129.98, 127.93, 126.89, 124.62, 114.04, 63.76, 49.12, 46.06, 45.52, 37.68, 34.30, 28.88, 28.77, 25.36, 24.81, 24.19, 23.07. HRMS (ESI) *m*/*z*: 397.1809 [M + H^+^], calcd. for C_24_H_28_ClNO_2_ 398.1893.

*5,5,9,9-Tetramethyl-3-(3-(4-methoxyphenyl)acryloyl)-4,5,6,7,8,9-hexahydro-5a,8-methanobenzo[d]azepin-2(3H)-one (***E16***).* White solid, yield 68%. mp. 122.2—123.1 °C; ^1^H NMR (600 MHz, CDCl_3_) δ: 7.70 (d, *J* = 15.5 Hz, 1H), 7.53 (d, *J* = 8.7 Hz, 2H), 7.21 (d, *J* = 15.5 Hz, 1H), 6.90 (d, *J* = 8.8 Hz, 2H), 5.77 (s, 1H), 4.12 (d, *J* = 14.4 Hz, 1H), 3.85 (s, 3H), 3.59 (d, *J* = 14.4 Hz, 1H), 1.98 (s, 1H), 1.93 (td, *J* = 12.4, 3.3 Hz, 1H), 1.85–1.79 (m, 1H), 1.74 (d, *J* = 10.2 Hz, 1H), 1.57 (tdd, *J* = 12.4, 5.9, 3.9 Hz, 1H), 1.42 (d, *J* = 10.1 Hz, 1H), 1.36–1.29 (m, 1H), 1.19 (s, 3H), 1.11 (s, 3H), 1.07 (s, 3H), 0.98 (s, 3H). ^13^C NMR (150 MHz, CDCl_3_) δ: 176.31, 170.18, 170.03, 161.08, 142.78, 129.91, 128.05, 119.66, 114.31, 114.15, 63.69, 55.35, 49.17, 46.07, 45.44, 37.69, 34.36, 28.91, 28.75, 25.38, 24.82, 24.20, 23.14. HRMS (ESI) *m*/*z*: 393.2304 [M + H^+^], calcd. for C_25_H_31_NO_3_ 394.2387.

*5,5,9,9-Tetramethyl-3-(3-(3,4-dimethoxyphenyl)acryloyl)-4,5,6,7,8,9-hexahydro-5a,8-methanobenzo[d]azepin-2(3H)-one (***E17***).* White solid, yield 35%. mp. 140.4—140.9 °C; ^1^H NMR (600 MHz, CDCl_3_) δ: 7.68 (d, *J* = 15.5 Hz, 1H), 7.22 (d, *J* = 15.5 Hz, 1H), 7.15 (dd, *J* = 8.3, 2.0 Hz, 1H), 7.10 (d, *J* = 2.0 Hz, 1H), 6.86 (d, *J* = 8.3 Hz, 1H), 5.77 (s, 1H), 4.11 (d, *J* = 14.4 Hz, 1H), 3.93 (d, *J* = 7.5 Hz, 6H), 3.61 (d, *J* = 14.4 Hz, 1H), 1.98 (s, 1H), 1.92 (td, *J* = 12.4, 3.3 Hz, 1H), 1.85–1.79 (m, 1H), 1.73 (d, *J* = 7.9 Hz, 1H), 1.57 (tdd, *J* = 12.4, 5.9, 3.9 Hz, 1H), 1.42 (d, *J* = 8.7 Hz, 1H), 1.33 (tdd, *J* = 11.1, 6.5, 2.3 Hz, 1H), 1.18 (s, 3H), 1.11 (s, 3H), 1.07 (s, 3H), 0.99 (s, 3H). ^13^C NMR (150 MHz, CDCl_3_) δ: 176.38, 170.07, 170.02, 150.82, 149.09, 143.19, 128.32, 122.81, 119.78, 114.26, 110.92, 109.88, 63.69, 55.96, 55.94, 49.21, 46.07, 45.43, 37.73, 34.38, 28.93, 28.71, 25.38, 24.80, 24.17, 23.19. HRMS (ESI) *m*/*z*: 423.2410 [M + H^+^], calcd. for C_26_H_33_NO_4_ 424.2489.

*5,5,9,9-Tetramethyl-3-(3-(pyridin-3-yl)acryloyl)-4,5,6,7,8,9-hexahydro-5a,8-methanobenzo[d]azepin-2(3H)-one (***E18***).* White solid, yield 27%. mp. 115.1—115.8 °C; ^1^H NMR (600 MHz, CDCl_3_) δ: 8.77 (d, *J* = 2.2 Hz, 1H), 8.58 (dd, *J* = 4.8, 1.6 Hz, 1H), 7.90 (d, *J* = 8.0 Hz, 1H), 7.65 (d, *J* = 15.6 Hz, 1H), 7.35 (d, *J* = 15.6 Hz, 1H), 7.31 (dd, *J* = 7.9, 4.8 Hz, 1H), 5.77 (s, 1H), 4.14 (d, *J* = 14.5 Hz, 1H), 3.58 (d, *J* = 14.4 Hz, 1H), 1.99 (s, 1H), 1.94 (td, *J* = 12.4, 3.3 Hz, 1H), 1.85–1.80 (m, 1H), 1.74 (dt, *J* = 7.8, 2.2 Hz, 2H), 1.58 (tdd, *J* = 12.5, 5.9, 3.9 Hz, 1H), 1.43 (d, *J* = 10.2 Hz, 1H), 1.33 (dddd, *J* = 12.5, 9.0, 5.9, 2.1 Hz, 1H), 1.19 (s, 3H), 1.12 (s, 3H), 1.08 (s, 3H), 0.99 (s, 3H). ^13^C NMR (150 MHz, CDCl_3_) δ: 177.12, 170.02, 169.35, 150.48, 149.92, 138.41, 134.25, 131.07, 124.25, 123.59, 113.96, 63.78, 49.12, 46.05, 45.58, 37.67, 34.29, 28.86, 28.79, 25.35, 24.80, 24.22, 23.07. HRMS (ESI) *m*/*z*: 364.2151 [M + H^+^], calcd. for C_23_H_28_N_2_O_2_ 365.2230.

*5,5,9,9-Tetramethyl-3-(3-(3,4-difluorophenyl)acryloyl)-4,5,6,7,8,9-hexahydro-5a,8-methanobenzo[d]azepin-2(3H)-one (***E19***).* White solid, yield 58%. mp. 150.2—150.3 °C; ^1^H NMR (600 MHz, CDCl_3_) δ: 7.57 (d, *J* = 15.5 Hz, 1H), 7.42–7.37 (m, 1H), 7.31–7.27 (m, 2H), 7.20 (d, *J* = 15.5 Hz, 1H), 7.18–7.13 (m, 1H), 5.76 (s, 1H), 4.14 (d, *J* = 14.4 Hz, 1H), 3.57 (d, *J* = 14.4 Hz, 1H), 1.99 (s, 1H), 1.94 (td, *J* = 12.4, 3.3 Hz, 1H), 1.86–1.79 (m, 1H), 1.74 (d, *J* = 7.9 Hz, 1H), 1.61–1.54 (m, 1H), 1.43 (d, *J* = 10.1 Hz, 1H), 1.33 (ddd, *J* = 12.7, 9.5, 4.3 Hz, 1H), 1.19 (s, 3H), 1.12 (s, 3H), 1.08 (s, 3H), 0.98 (s, 3H). ^13^C NMR (150 MHz, CDCl_3_) δ: 177.05, 170.04, 169.46, 139.96, 132.57, 124.84, 124.81, 124.79, 123.24, 117.63, 117.51, 116.42, 116.30, 114.00, 63.76, 49.10, 46.06, 45.57, 37.66, 34.29, 28.86, 28.80, 25.35, 24.80, 24.22, 23.06. HRMS (ESI) *m*/*z*: 399.2010 [M + H^+^], calcd. for C_24_H_27_F_2_NO_2_ 400.2091.

### 4.2. Cell Culture and Cell Cytotoxicity Assay

#### Cell Culture and CCK-8 Assay

Three cancer cell lines (human breast adenocarcinoma cells MCF-7, human lung adenocarcinoma epithelial cells A549, and human hepatocellular carcinoma cells HepG2) were obtained from the Cell Bank of the Chinese Academy of Sciences (Shanghai, China). LO2 human normal liver cells were also purchased from STEM RECELL (Beijing, China, STM-CL-5770). The anti-proliferation activity of compounds was tested on three cancer cell lines. The cytotoxicity of compounds as tested on normal cell line (human normal liver cells LO2). Cells were seeded at a density of 5000 cells per well in 96-well plates. Natural product piperlongumine was used as the positive control. All experiments were performed in triplicate. Three cancer cell lines were cultured in High DMEM medium or RIPM 1640 containing 10% FBS (Sijiqing, Hangzhou, China) and 1% antibiotics at 37 °C in a 5% CO_2_ incubator. The experimental cells were seeded in a 96-well plate at 5000 cells per well and incubated for 24 h. The old medium was replaced with experimental medium, and the cells were cultured for 48 h. Afterward, 10 µL of CCK-8 solution was added to each well, and the cells were incubated for 3 h. The absorbance at 450 nm was measured using a microplate reader.

### 4.3. Anticancer Mechanism Studies

#### 4.3.1. Cell Apoptosis Assay

MCF-7 cells were seeded at a density of 6 × 10^5^ cells per well in 12-well plates and incubated in a 37 °C, 5% CO_2_ incubator for 24 h. After treatment with different concentrations of compound **E10**, the cells were washed with PBS two times, collected by trypsin enzymic digestion and centrifugation, and the collected cells were subsequently stained with 5 µL Annexin V-APC (YSRIBIO-C1084) and 5 µL PI for 10 min in the dark conditions. The cell apoptosis was measured with the flow cytometry (BD), and the data were analyzed by flowjo 10.6 software.

#### 4.3.2. Cell Cycle Assay

For cell cycle analysis, MCF-7 cells were seeded at a density of 2 × 10^5^ cells per well in 6-well plates. MCF-7 cells were treated with different concentrations of compound **E10** for 24 h. After that, the cells were washed with PBS twice, collected by trypsin enzymic digestion and centrifugation, and the collected cells were subsequently fixed with 70% ice-cold ethanol overnight at 4 °C. The cells were stained with PI (50 μg/mL) the next day. The cell cycle distributions were measured through flow cytometry (BD), and the data were analyzed by Modfit 5.1 software.

#### 4.3.3. Monodansylcadaverine (MDC) Staining Assay

MDC is a staining indicator for the formation of autophagosome. MCF-7 cells were seeded at a density of 2 × 10^5^ cells per well in 6-well plates. MCF-7 cells were inoculated into a 6-well plate, and the cells were treated with **E10** for 24 h. The treated cells were washed twice with buffer and then stained with MDC (Beijing Solarbio Science & Technology Co., Ltd., Beijing, China) for 40 min. The cells were then detected under a fluorescence microscope (Carl Zeiss, Jena, Germany).

#### 4.3.4. Intracellular ROS Assay

For the detection of intracellular ROS levels, MCF-7 cells were seeded at a density of 5 × 10^5^ cells per well in a 12-well plate and treated with different concentrations of compound **E10** for 24 h. The cells were collected and stained with 5 μM of DCFH-DA dye (Yeasen, Shanghai, China, 50101ES01) in the incubator for 30 min. Flow cytometry (BD) detected the stained cell samples, and the data were analyzed using FlowJo software.

#### 4.3.5. MMP Assay

MCF-7 cells were treated with different concentrations of compound **E10** for 24 h. After that, the cells were collected and stained with JC-1 (Yeasen, Shanghai, China, 40706ES60) for 15 min. The cell samples were detected by flow cytometry (BD) and the data were analyzed by flowjo 10.6 software.

#### 4.3.6. Comet Assay

MCF-7 cells were seeded in 24-well plastic plates at a density of 5 × 10^4^ cells per well and treated with compound **E10** for 24 h. The alkaline DNA comet assay was performed according to the instructions of the OxiSelectTM Comet Assay Kit (Cell Biolabs, San Diego, CA, USA, Cat. # STA-350) and analyzed using a Zeiss fluorescent microscope (Jena, Germany). The free software Casplab-1.2.3b2 (CAS PLab, Wroclaw, Poland) was used to calculate the percentage of tail DNA and Olive Tail Moment (OTM) as markers of DNA damage (Olive Tail Moment = Tail DNA (%) × Tail Moment Length).

#### 4.3.7. Western Blotting Analysis

MCF-7 exponentially growing cells were plated in 6-well plates (2 × 10^5^ cells/well). Then, derivatives **E10** (0.5, 1.0 and 2.0 μM) were added to the well. After incubation for an additional 48 h, the cell culture medium was removed, and the cells were washed twice with ice-cold PBS. Subsequently, the cells were lysed to extract the total protein and centrifuged at 12,000 rpm for 5 min at 4 °C using lysis buffer (Beyotime, Shanghai, China, Cat. # P0013E). All protein concentrations were determined using a BCA kit. Equal amounts of protein (20–30 µg per lane) were loaded onto the gel. An equal amount of protein was analyzed using 12% SDS-PAGE gels and transferred to Hybond-P membrane containing polyvinylidene difluoride (PVDF). The membranes were then blocked with BSA for 2 h at room temperature and incubated with primary antibodies against p-mTOR (ImmunoWay Biotechnology, Beijing, China, YP1444, 1:5000), P62 (Proteintech Group, Inc., Wuhan, China, 60268-1-IG, 1:5000), LC3-I/II (Proteintech Group, Inc., Wuhan, China, 10253-2-AP, 1:5000) and p-AMPK, AMPK, Bcl-2, Bax, Cleaved-caspase 7 (ImmunoWay Biotechnology, Beijing, China, YP0251, 1:5000) overnight at 4 °C and with secondary antibodies (AP-conjugated goat anti-rabbit or anti-mouse, 1:10,000) for 2 h. Densitometry was performed in ImageJ 2 within the linear range. Targets were normalized to β-actin (or to the corresponding total protein for phospho-target), then expressed relative to vehicle (0.1% DMSO = 1.0). Where indicated, ratios such as LC3-II/LC3-I and Bax/Bcl-2 were calculated per lane before statistics.

#### 4.3.8. Autophagy Assay Using Transmission Electron Microscopy

MCF-7 cells were treated with **E10** for 24 h. Cells were washed twice with pre-cold PBS, fixed in 2% glutaraldehyde at 4 °C for 2 h. Then, the sample was post-fixed in 2% osmium tetroxide for 1 h at 4 °C in the dark. Samples were dehydrated through a graded ethanol series, infiltrated and embedded in Spurr’s resin, and polymerized at 60 °C overnight. Ultrathin sections (~70–90 nm) were cut on an ultramicrotome, mounted on copper grids, counterstained with uranyl acetate and lead citrate, and examined by transmission electron microscopy (JEM-100CX; JEOL, Tokyo, Japan) at the indicated magnifications.

### 4.4. Three-Dimensional Cell Culture

Malignant glioblastoma MCF-7 cells were seeded into cell-repellent 96-well U-bottom plates (Sbio, Tokyo, Japan) at a density of 3000 cells per well and cultured for 72 h to promote spheroid formation. Subsequently, 50 μL of the culture medium was carefully removed and replaced with 50 μL of **E10**-containing medium. The spheroids were then incubated for an additional 96 h. After treatment, spheroids were stained with 5 μM PI and 10 μM Calcein-AM, and observed under a fluorescence microscope (Olympus, Tokyo, Japan).

### 4.5. Anticancer Activity in Zebrafish Models

All experimental procedures were approved by ACUC of Shandong Academy of Sciences (Animal Care and Use Committee). Wild-type AB of zebrafish were obtained from Biology Institute, Qilu University of Technology. The embryos were incubated in special embryonic medium (0.17 mM KCl, 5.0 mM NaCl, 0.4 mM CaCl_2_, and 0.16 mM MgSO_4_) at 28 °C. 48 h-old zebrafish embryos were microinjected with CM-Dil labelled MCF-7 cells under stereomicroscopy. After injection, zebrafish embryos with the same injection spot were randomly planted on a six-well plate. These xenograft zebrafish were sorted into four groups of about 15–20 zebrafish each and treated with 0.5, 1.0, 2.0 μg/mL of compound **E10** for 48 h, 1.0 μM doxorubicin as a positive control group. After treatment, the fluorescence intensity of living cells was examined with laser confocal microscopy (Olympus, Tokyo, Japan).

### 4.6. Statistical Analysis

For the analysis of data obtained in the in vivo, ex vivo tests, GraphPad Prism software (v. 9.0, CA, USA) was used. Numerical results from the experiments were expressed as the mean ± standard error of the mean (SEM). Repeated measures of analysis of variance (ANOVA), one-way ANOVA, followed by Dunnett’s post hoc test were used for the statistical analysis of the results. Significance is indicated as ns (not significant), * *p* < 0.05, ** *p* < 0.01, *** *p* < 0.001, or **** *p* < 0.0001. Exact n values and tests used are specified in the corresponding figure legends.

## Figures and Tables

**Table 1:** Antiproliferative activity of compounds **E1–E19** against various cell lines.

Compd.	R	IC_50_ ^*^ (µM)			
		MCF-7	HepG2	A549	LO2
**E1**	4-F-C_6_H_4_	9.18 ± 0.11	>20	11.73 ± 0.16	>20
**E2**	4-CH_3_-C_6_H_4_	1.29 ± 0.04	6.60 ± 0.07	3.18 ± 0.16	>20
**E3**	4-Cl-C_6_H_4_	18.97 ± 0.13	7.09 ± 0.33	16.93 ± 0.27	>20
**E4**	4-CF_3_-C_6_H_4_	16.74 ± 0.16	>20	14.98 ± 0.10	>20
**E5**	2-CH_3_-C_6_H_4_	2.86 ± 0.12	1.86 ± 0.09	3.89 ± 0.44	>20
**E6**	3-F-C_6_H_4_	5.32 ± 0.11	5.96 ± 0.05	5.34 ± 0.16	>20
**E7**	3-Cl-C_6_H_4_	2.67 ± 0.11	6.67 ± 0.06	2.28 ± 0.10	>20
**E8**	3-CH_3_-C_6_H_4_	1.38 ± 0.12	1.62 ± 0.10	3.71 ± 0.08	>20
**E9**	2-OCH_3_-C_6_H_4_	2.22 ± 0.07	2.17 ± 0.24	2.13 ± 0.16	>20
**E10**	3-CF_3_-C_6_H_4_	0.32 ± 0.05	1.36 ± 0.18	1.39 ± 0.32	14.41± 0.04
**E11**	2-CF_3_-C_6_H_4_	5.63 ± 0.30	4.53 ± 0.12	18.30 ± 0.21	>20
**E12**	3-Br-C_6_H_4_	1.26 ± 0.19	4.89 ± 0.52	3.55 ± 0.04	11.26 ± 0.06
**E13**	2-Br-C_6_H_4_	2.12 ± 0.04	2.67 ± 0.16	4.64 ± 0.06	>20
**E14**	4-Br-C_6_H_4_	1.27 ± 0.04	2.38 ± 0.16	3.21 ± 0.16	>20
**E15**	2-Cl-C_6_H_4_	1.58 ± 0.16	2.09 ± 0.16	3.47 ± 0.16	>20
**E16**	2-OCH_3_-C_6_H_4_	2.67 ± 0.18	2.44 ± 0.16	5.28 ± 0.10	>20
**E17**	3,4-OCH_3_-C_6_H_4_	2.16 ± 0.03	2.56 ± 0.16	5.28 ± 0.08	>20
**E18**	3-pyridine	>20	>20	>20	>20
**E19**	3,4-F-C_6_H_4_	17.07 ± 0.06	18.43 ± 0.16	18.90 ± 0.16	>20
**PL**	−	2.79 ± 0.43	3.46 ± 0.25	5.83 ± 0.26	−

* IC_50_ values are the means ± SD of three independent experiments.

## Data Availability

The original contributions presented in this study are included in the article/supplementary material. Further inquiries can be directed to the corresponding authors.

## Supplementary Materials

The following supporting information can be downloaded at: https://www.mdpi.com/article/10.3390/molecules30194013/s1.
