# Supplementary material for: Novel Isolongifolenone-Based Caprolactam Derivatives as Potential Anticancer Agents via the p53/mTOR/Autophagy Pathway
Source: Molecules. 2025 Oct 8;30(19):4013. doi: 10.3390/molecules30194013 (PMC12526212; doi:10.3390/molecules30194013)
Supplement: Supplementary file 1 [file molecules-30-04013-s001.zip › molecules-3781719-supplementary.pdf]

Novel Isolongifolenone-Based Caprolactam Derivatives as Potential Anticancer Agents via the p53/ mTOR/ Autophagy Pathway

Yunyun Wang [1*], Min Hu [1], Jiale Han[1],Yuxun Zhao [2], Biao Xiong [1], Peihai Li [3], Shifa Wang [2*]

[1] School of Pharmacy and Jiangsu Province Key Laboratory for Inflammation and Molecular Drug Target, Nantong University, Nantong 226001, China
[2] Co-Innovation Center of Efficient Processing and Utilization of Forest Resources, College of Chemical Engineering, Nanjing Forestry University, Nanjing 210037, China
[3] Engineering Research Center of Zebrafish Models for Human Diseases and Drug Screening of Shandong Province, Biology Institute, Qilu University of Technology (Shandong Academy of Sciences), Jinan 250103, China

[*]Corresponding author: Dr. Yunyun Wang and Prof. Shifa Wang
Email: wangyunyun91@ntu.edu (Y. Wang), wangshifa65@163.com (S. Wang)

1. ^{1}H NMR and ^{13}C NMR of compounds **E1-E19** S1-S38
2. HRMS of compounds **E1-E19** S39-S57

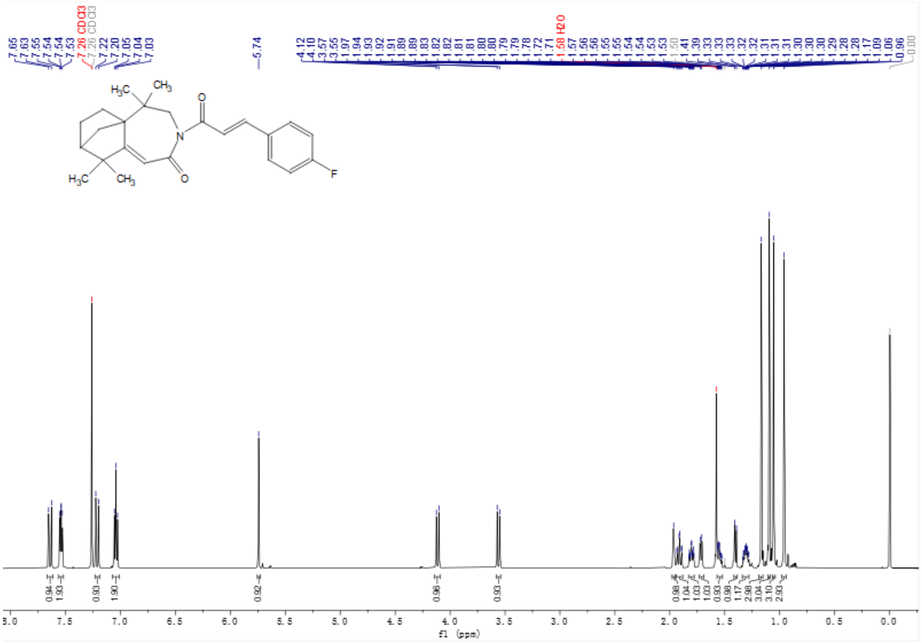

Figure S1: ^{1}H NMR of compound E1

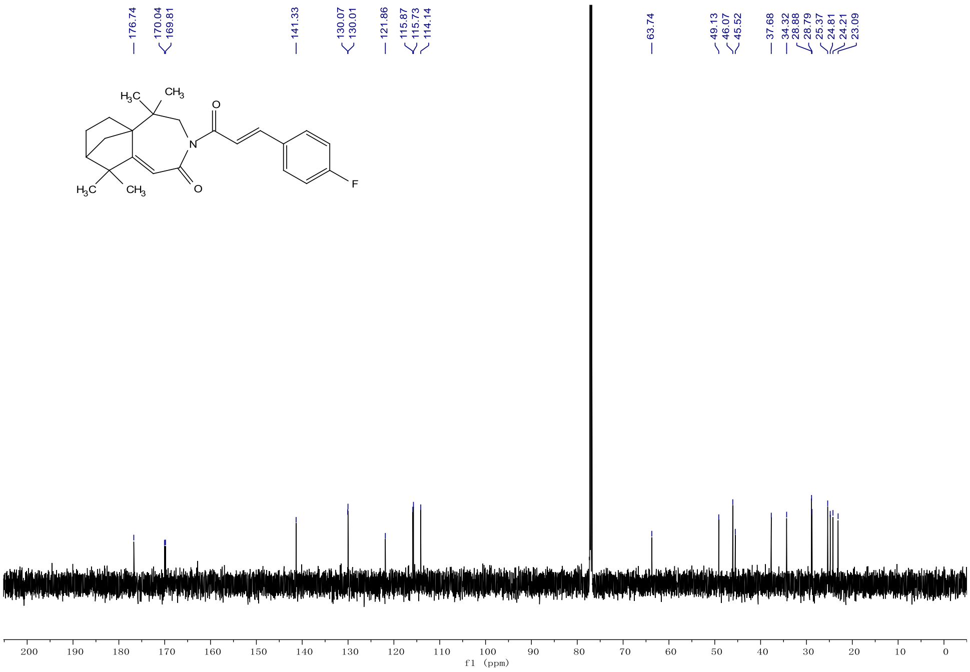

Figure S2: ^{13}C NMR of compound E1

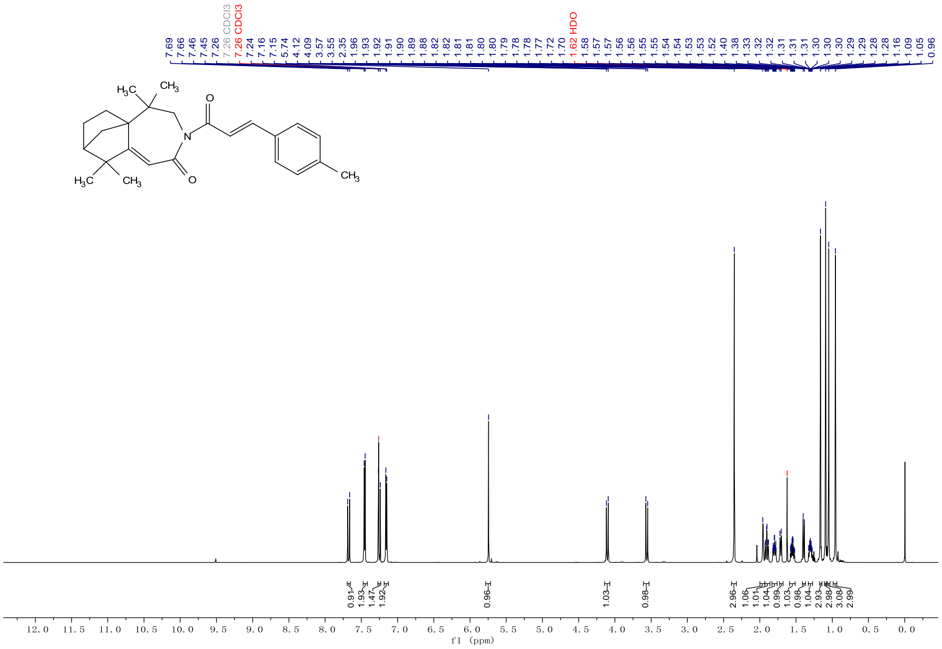

Figure S3: ^{1}H NMR of compound E2

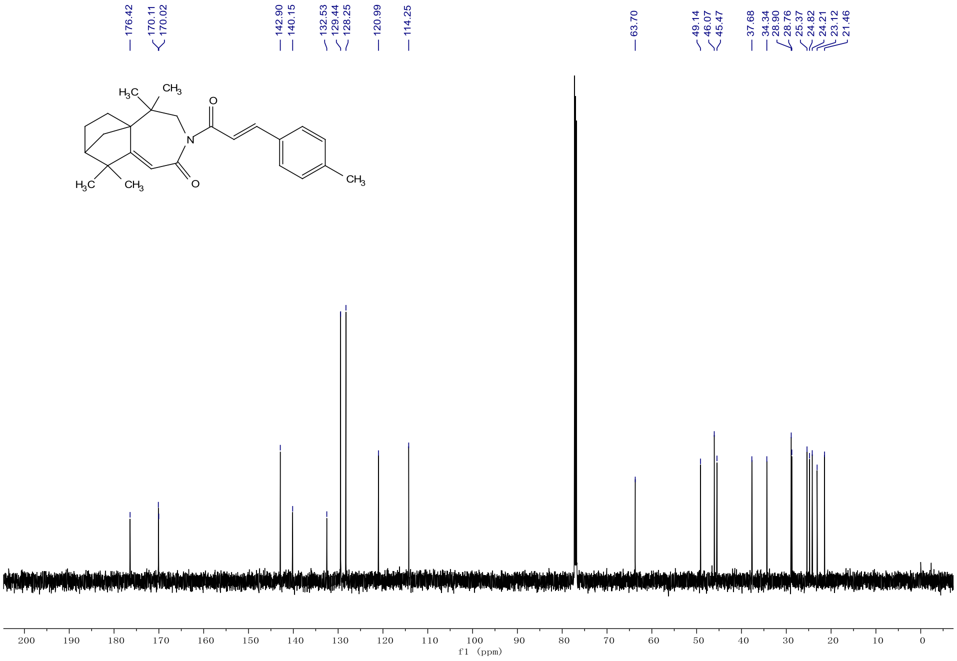

Figure S4: ^{13}C NMR of compound E2

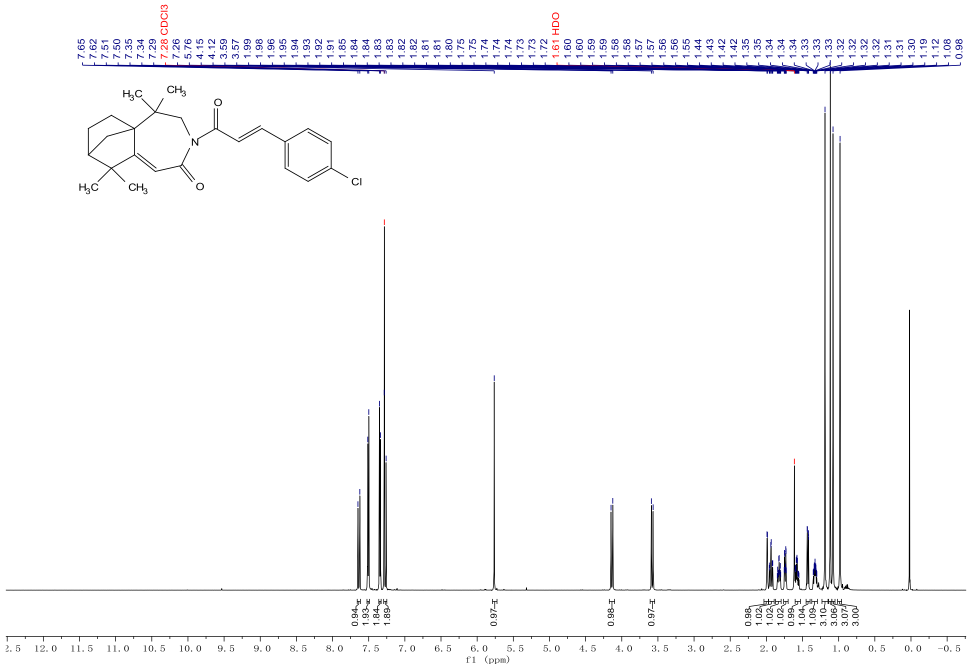

Figure S5: ^{1}H NMR of compound E3

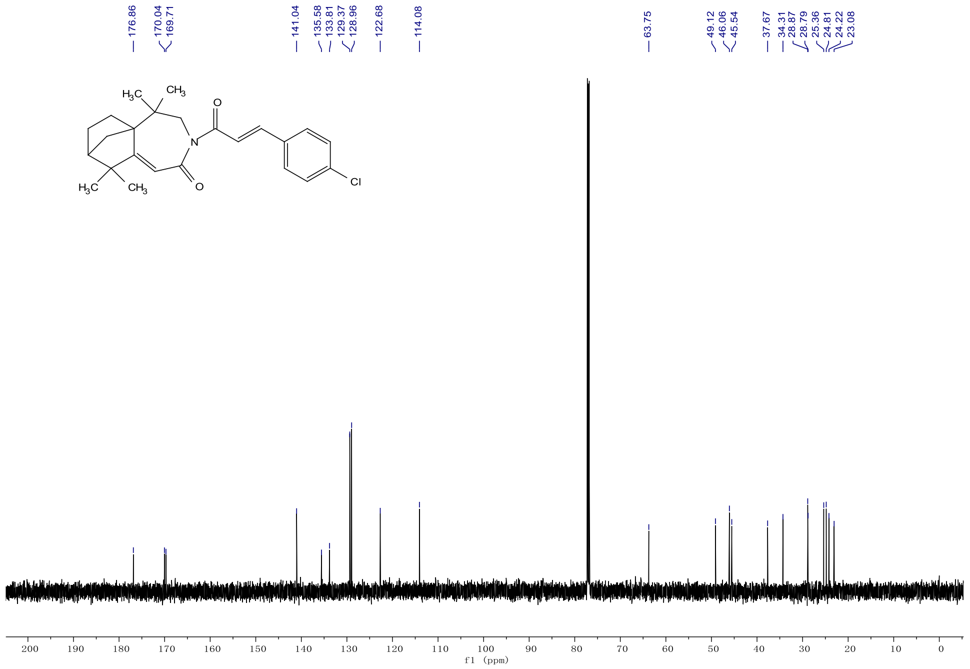

Figure S6: ^{13}C NMR of compound E3

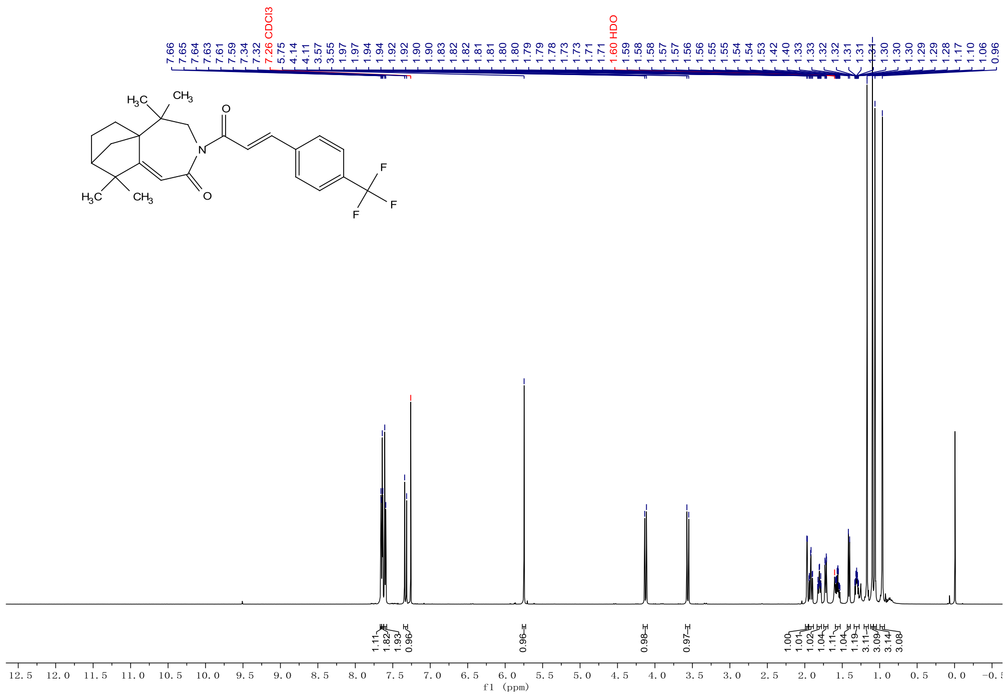

Figure S7: ^{1}H NMR of compound E4

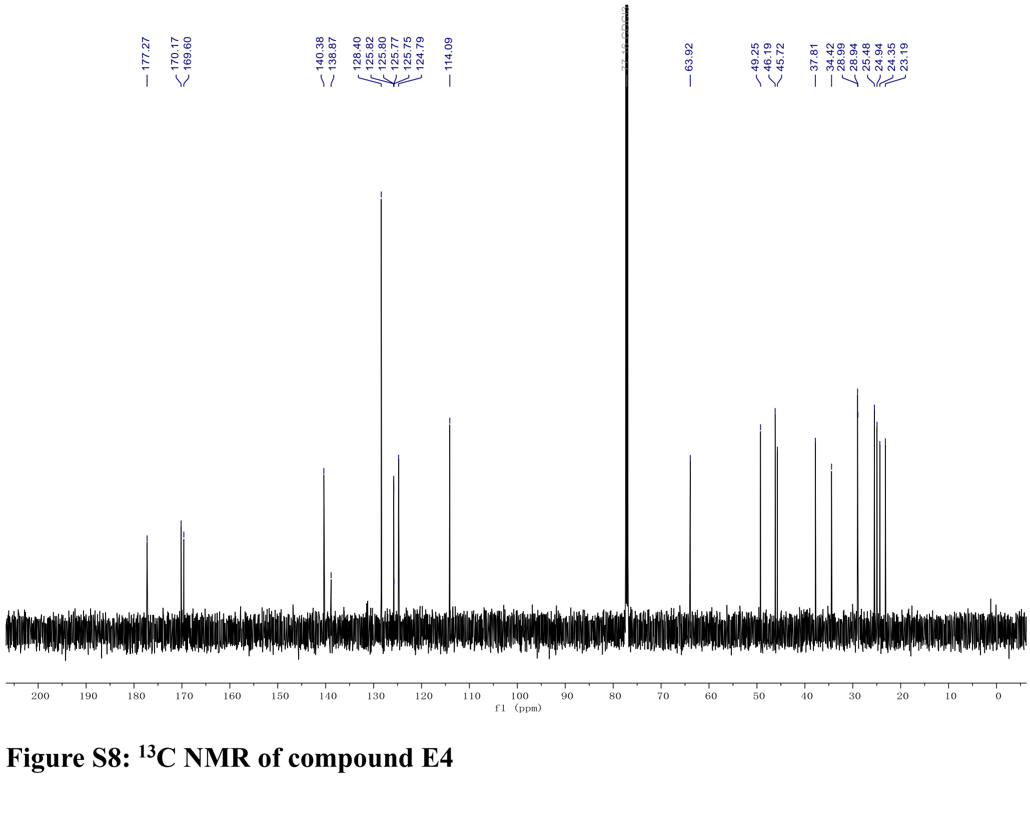

Figure S8: ^{13}C NMR of compound E4

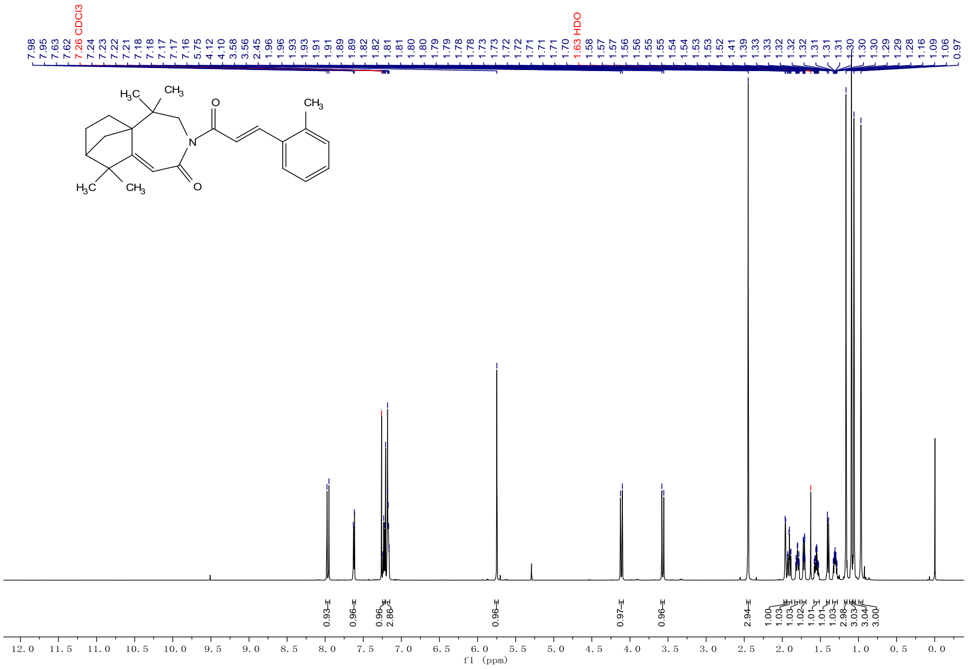

Figure S9: ^{1}H NMR of compound E5

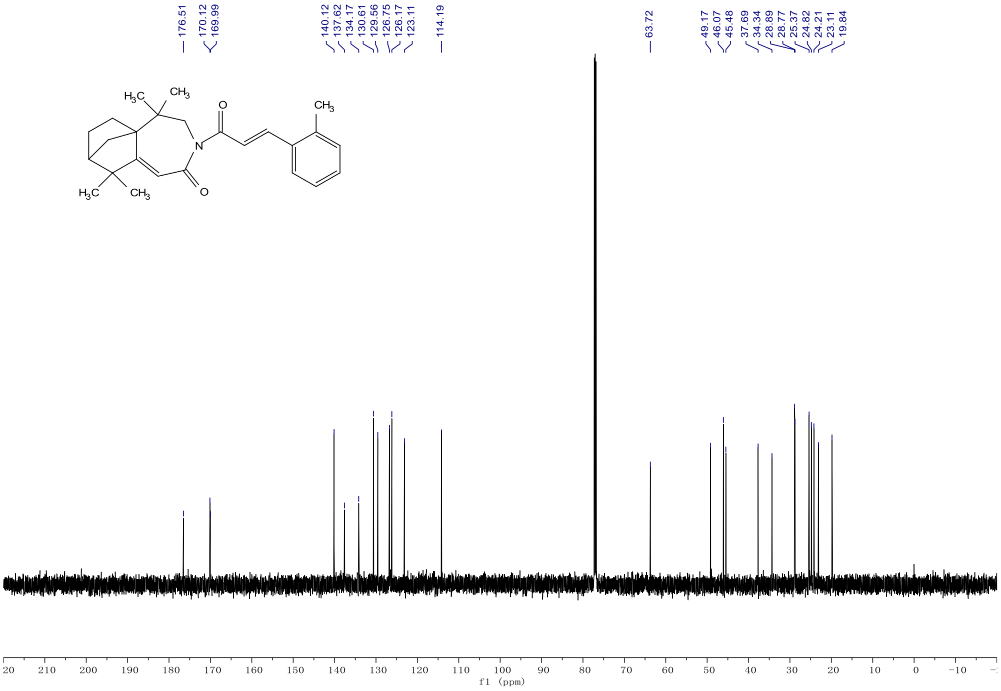

Figure S10: ^{13}C NMR of compound E5

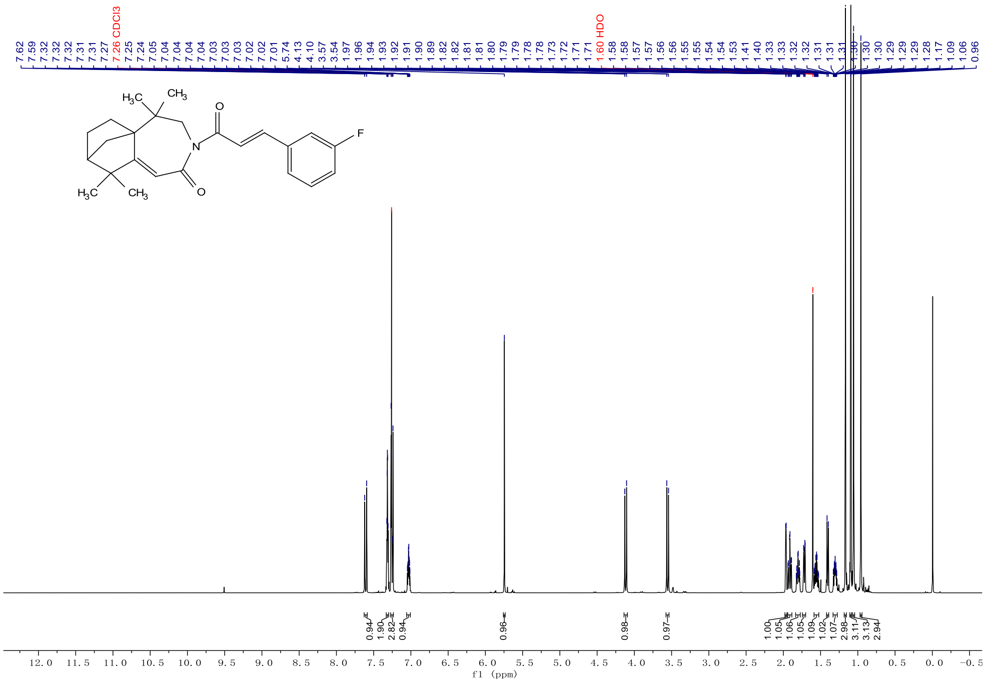

Figure S11: ^{1}H NMR of compound E6

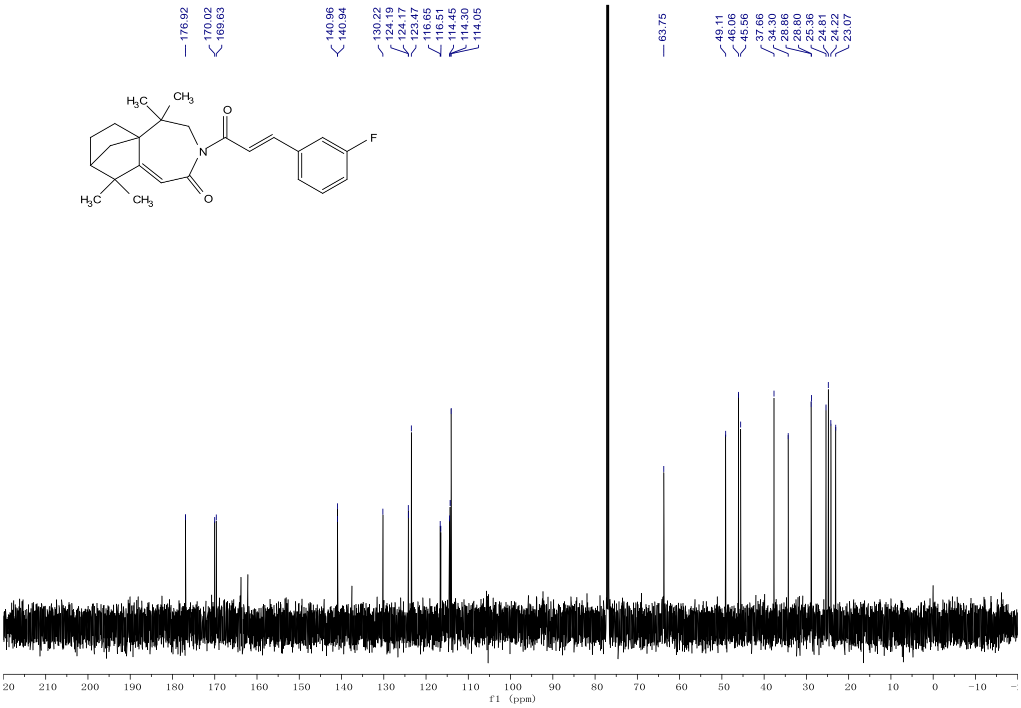

Figure S12: ^{13}C NMR of compound E6

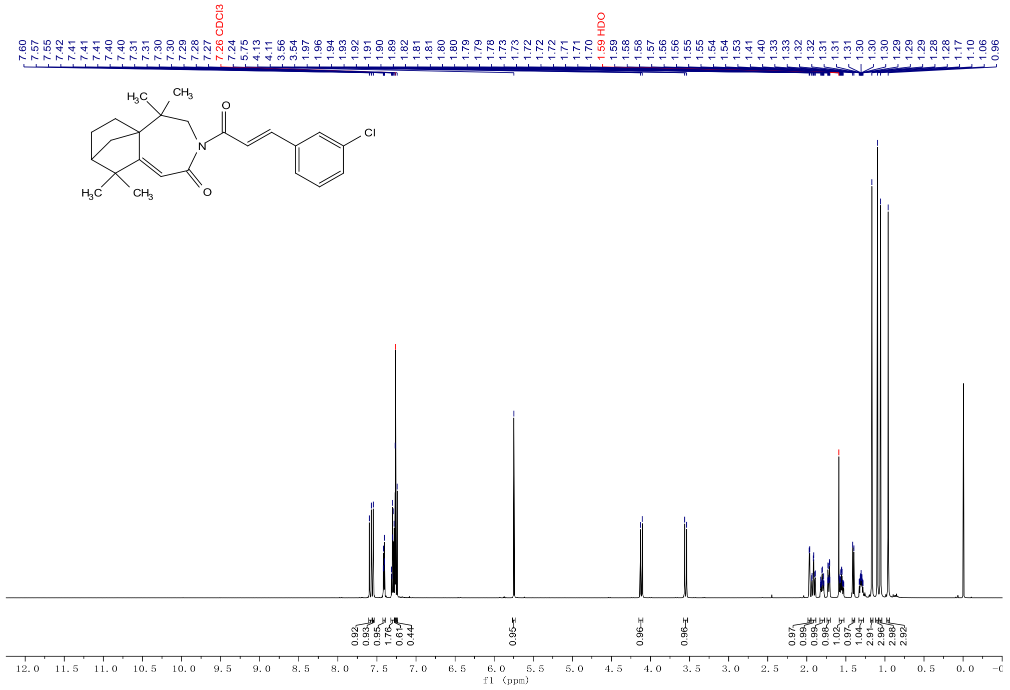

Figure S13: ^{1}H NMR of compound E7

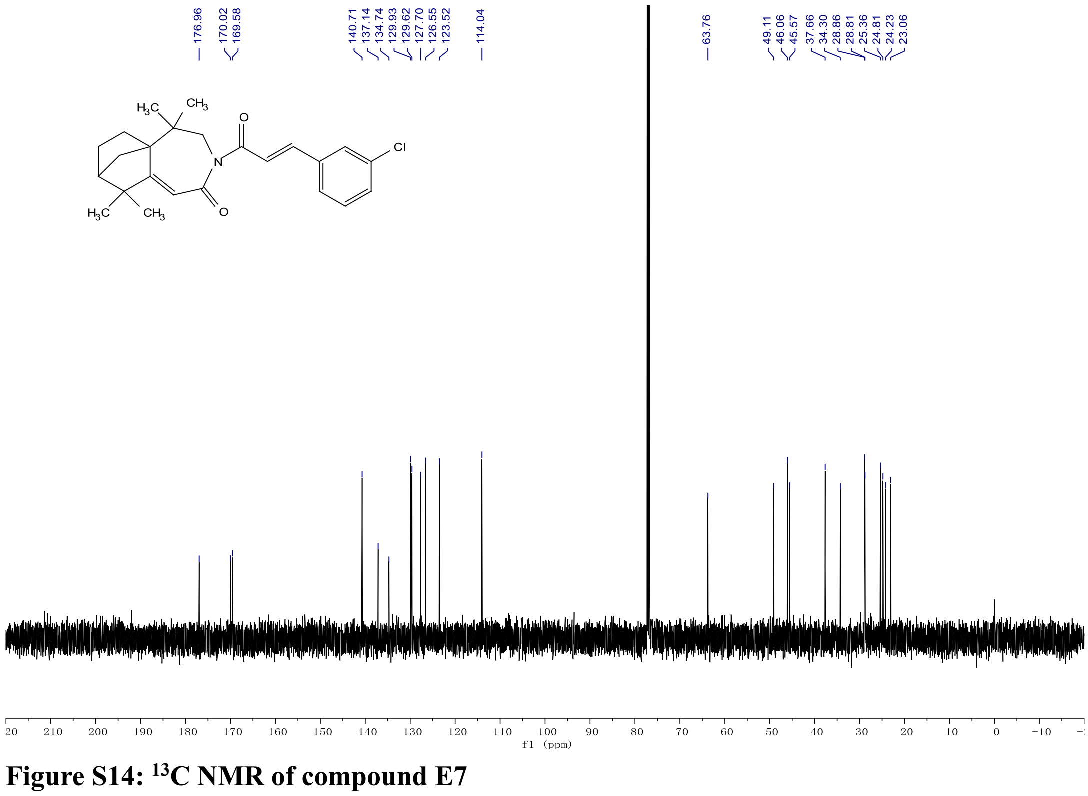

Figure S14: ^{13}C NMR of compound E7

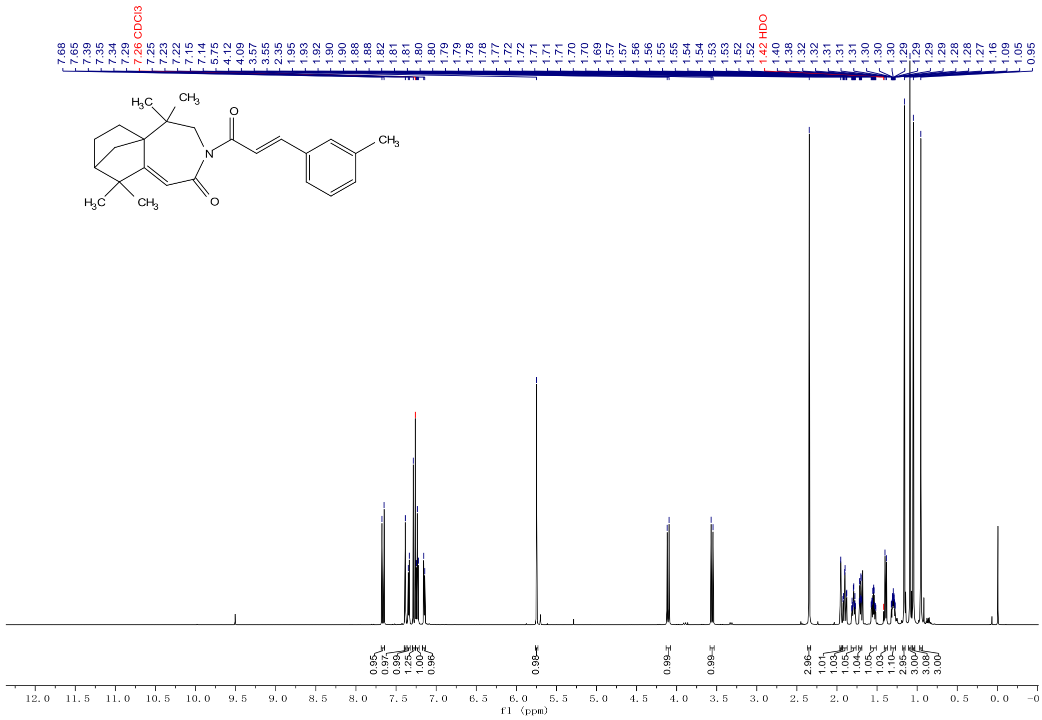

Figure S15: ^{1}H NMR of compound E8

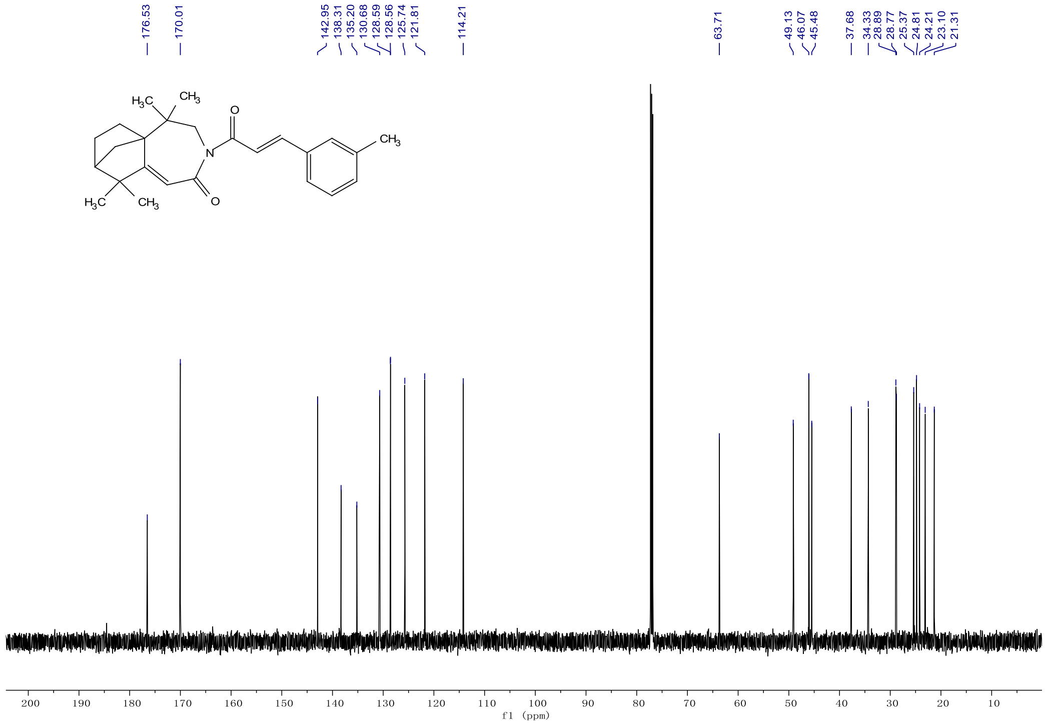

Figure S16: ^{13}C NMR of compound E8

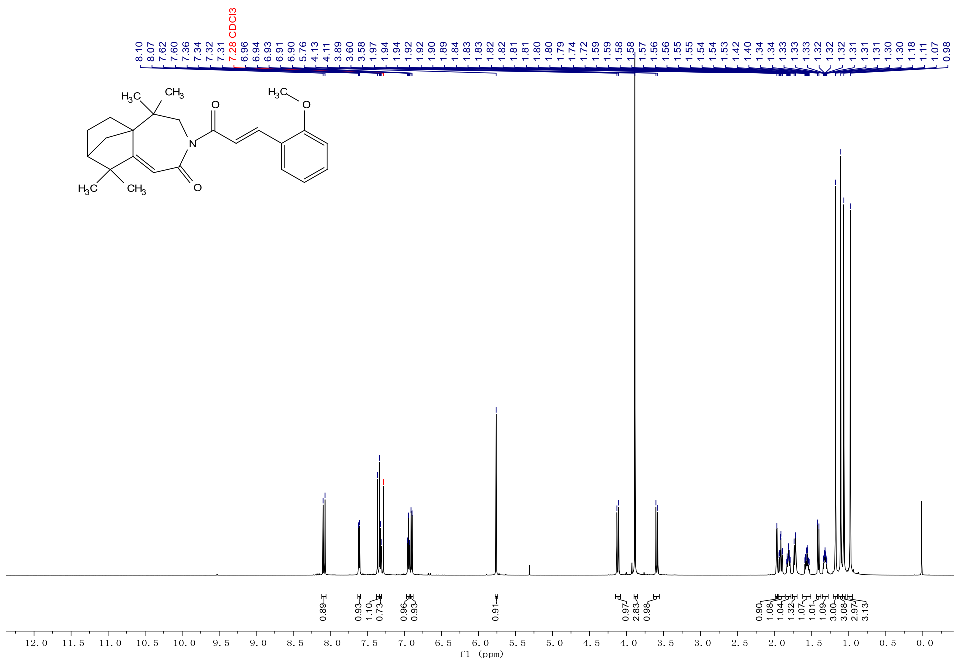

Figure S17: ^{1}H NMR of compound E9

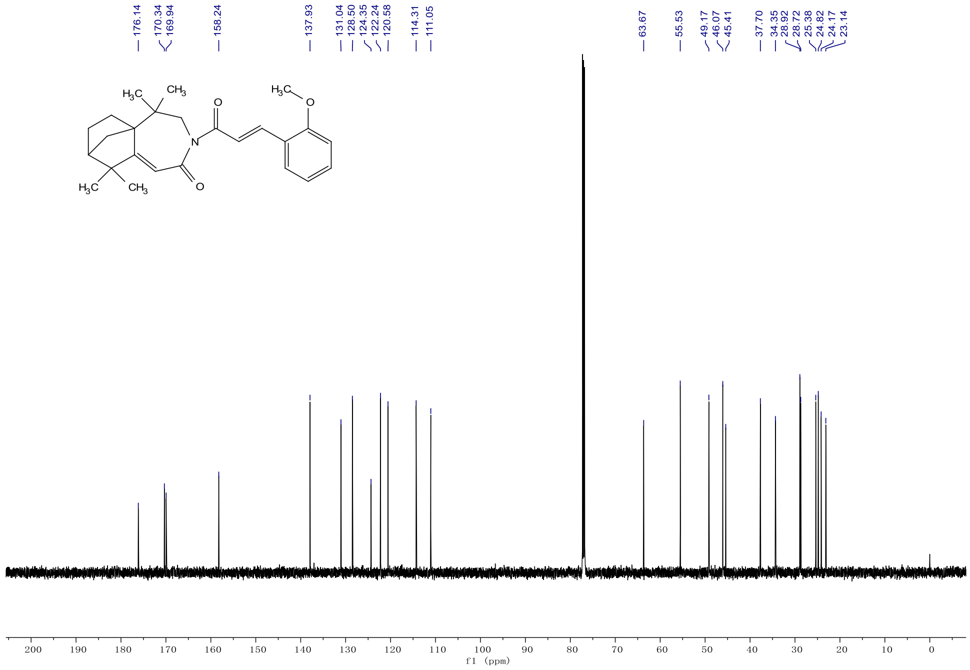

Figure S18: ^{13}C NMR of compound E9

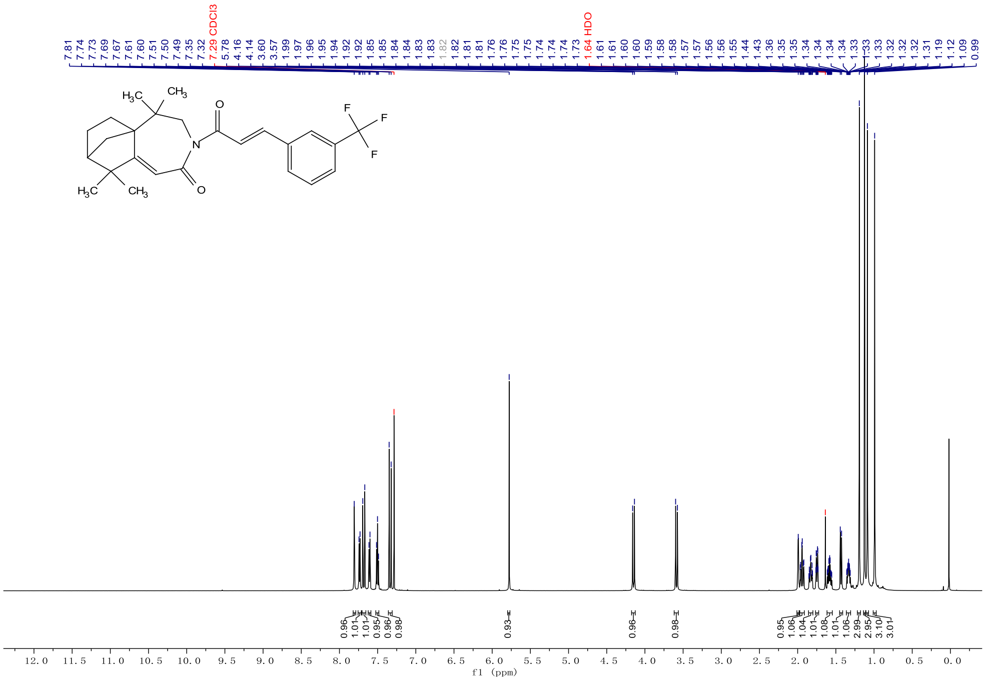

Figure S19: ^{1}H NMR of compound E10

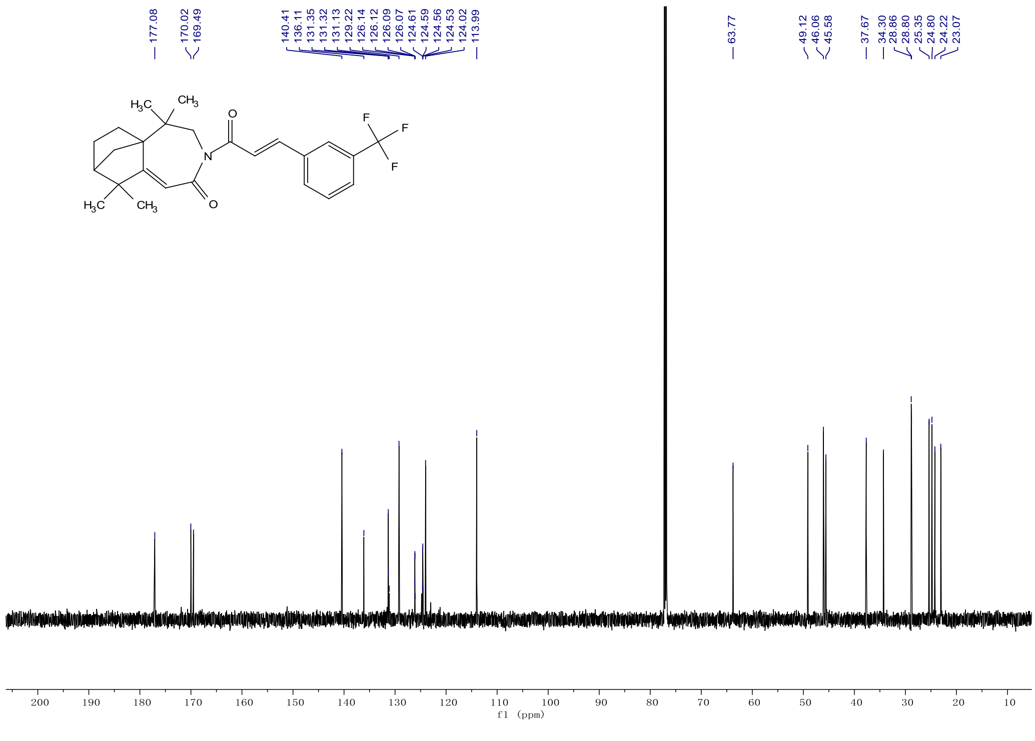

Figure S20: ^{13}C NMR of compound E10

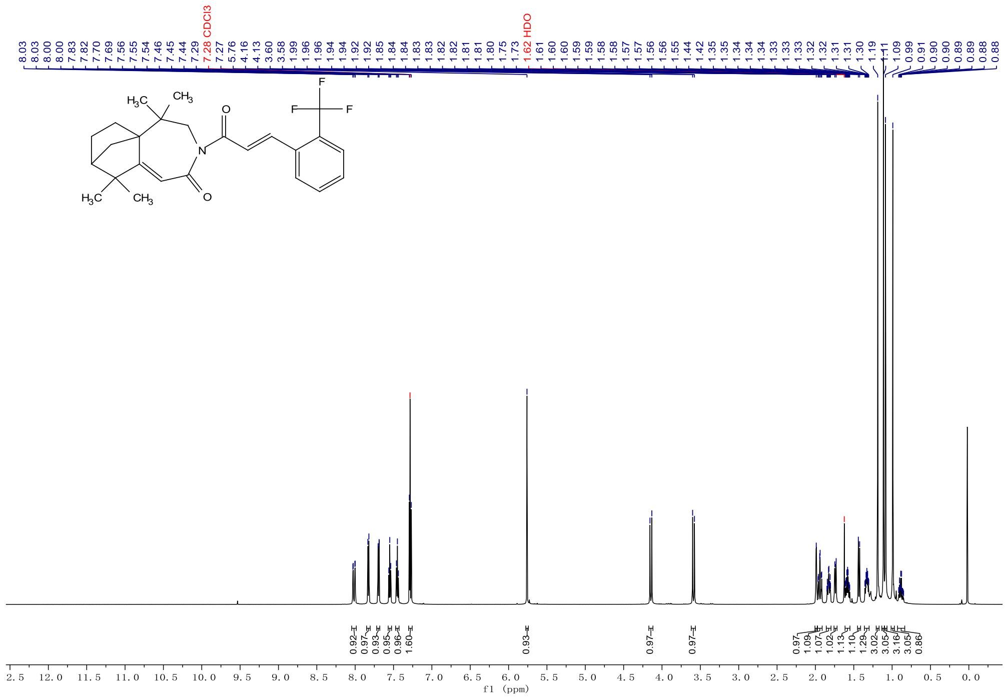

Figure S21: ^{1}H NMR of compound E11

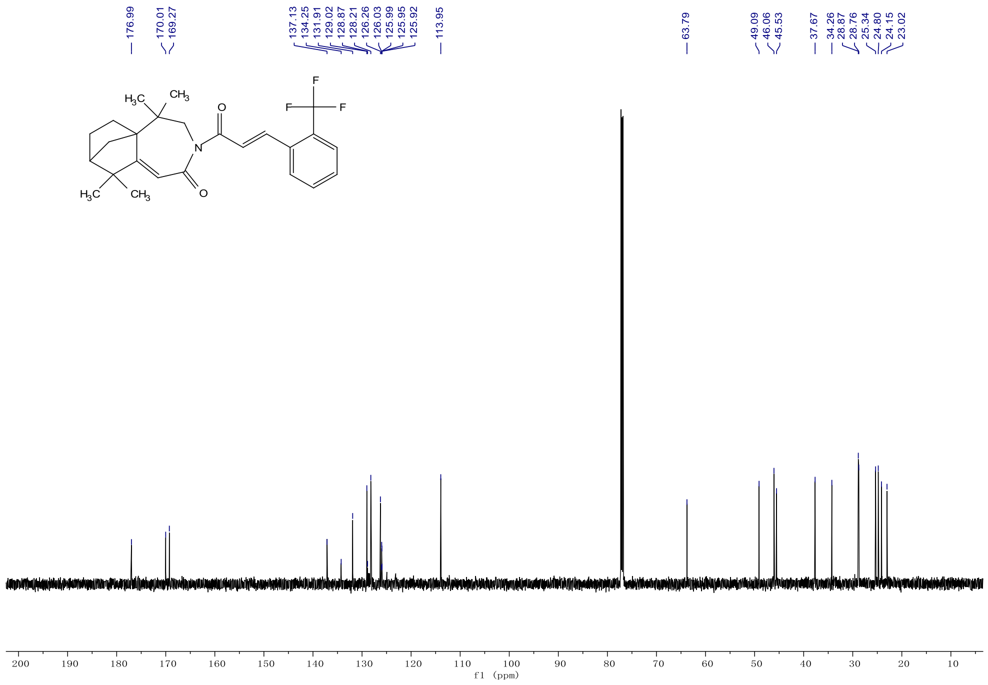

Figure S22: ^{13}C NMR of compound E11

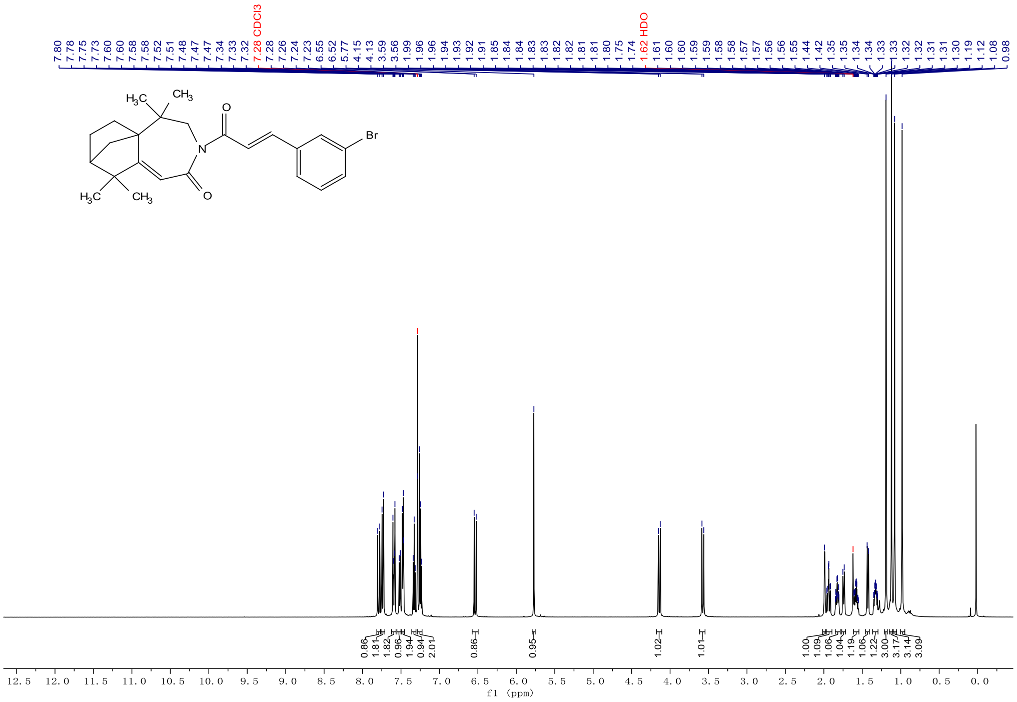

Figure S23: ^{1}H NMR of compound E12

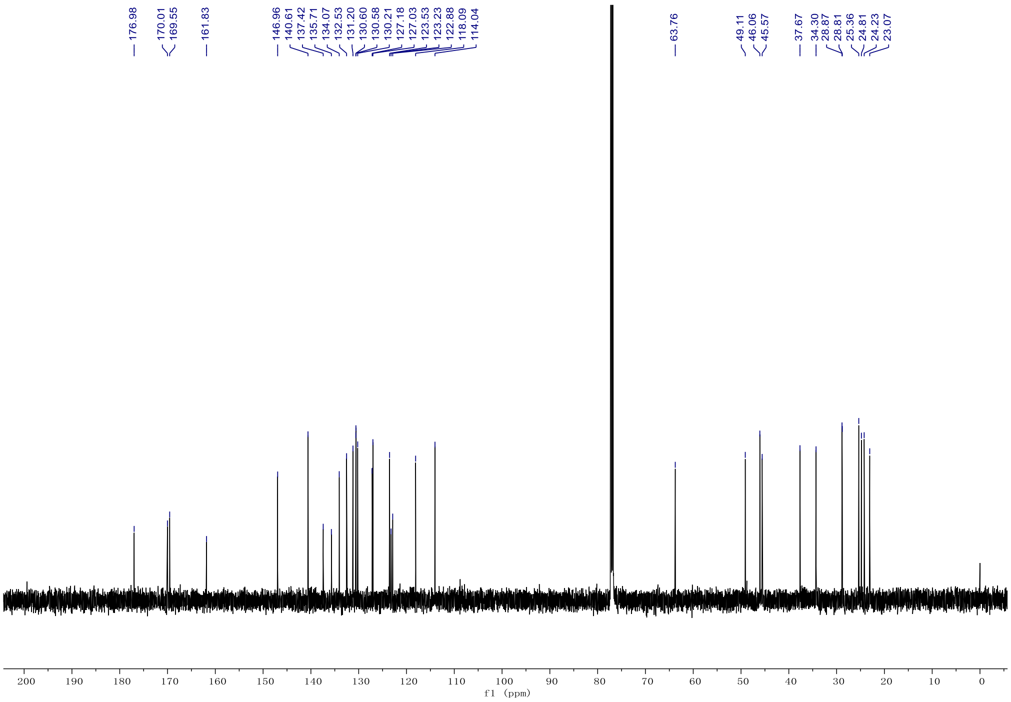

Figure S24: ^{13}C NMR of compound E12

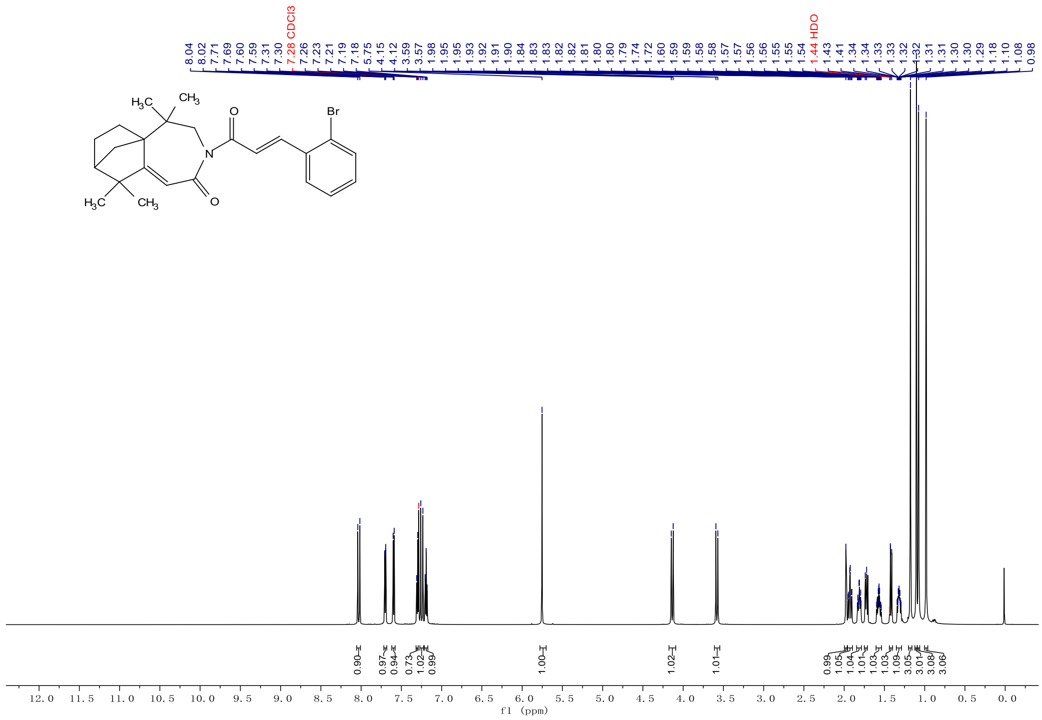

Figure S25: ^{1}H NMR of compound E13

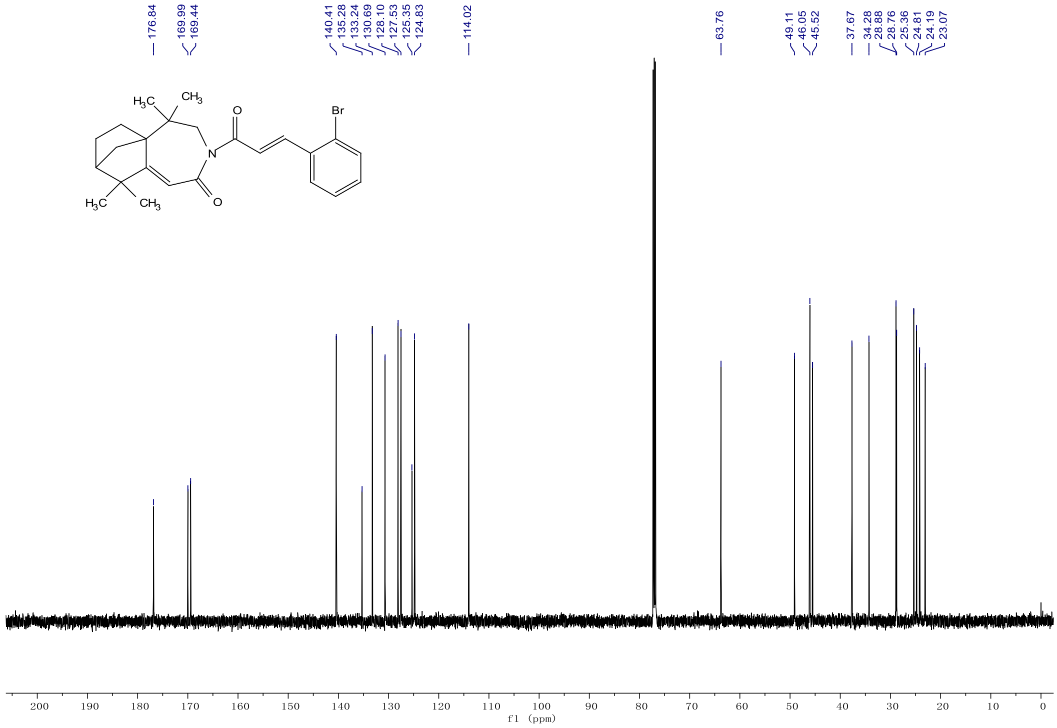

Figure S26: ^{13}C NMR of compound E13

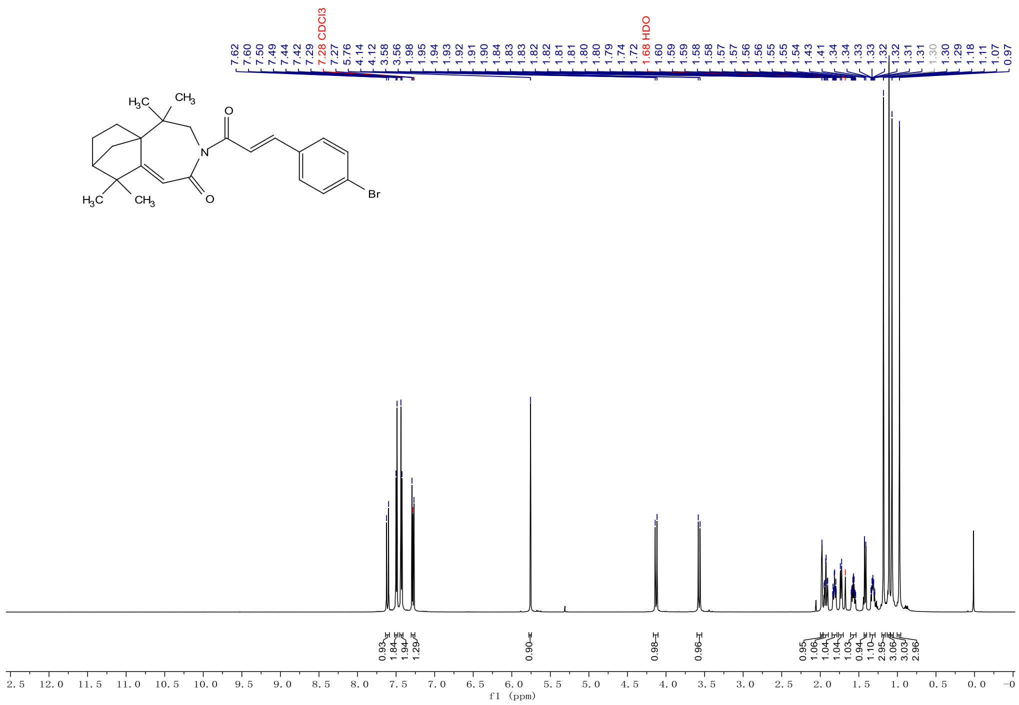

Figure S27: ^{1}H NMR of compound E14

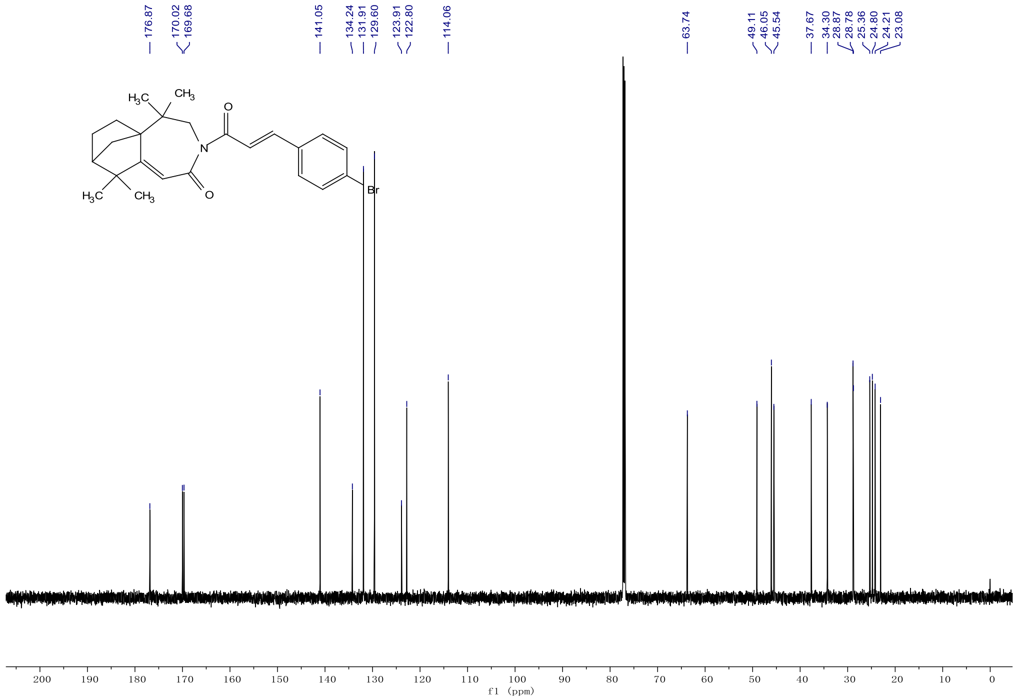

Figure S28: ^{13}C NMR of compound E14

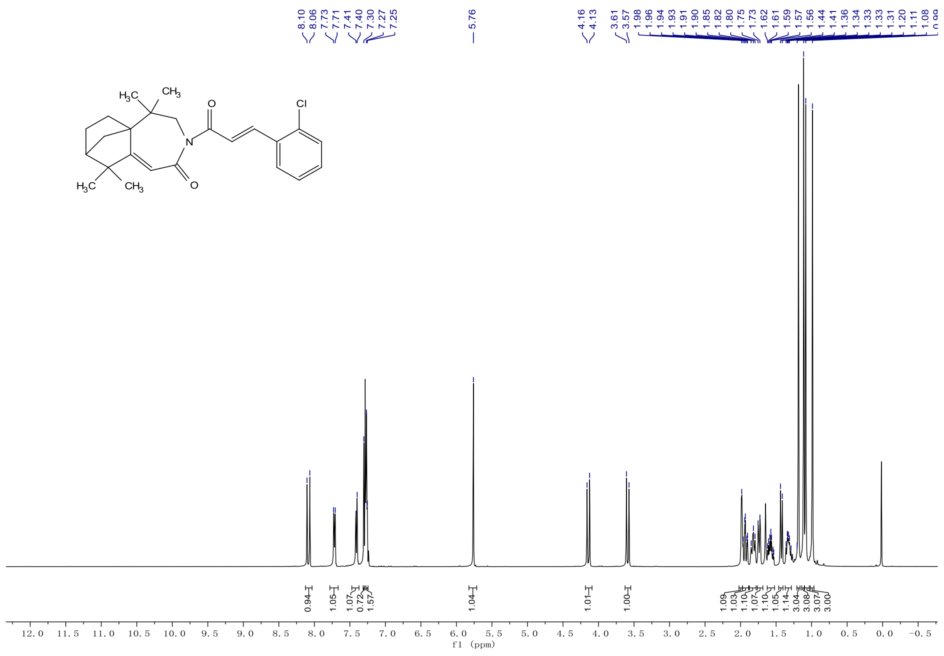

Figure S29: ^{1}H NMR of compound E15

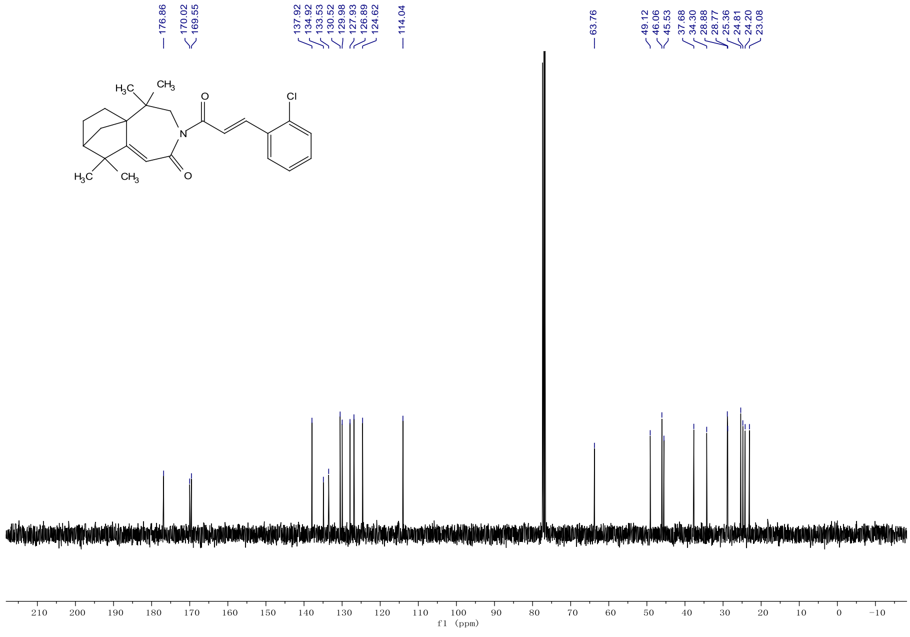

Figure S30: ^{13}C NMR of compound E15

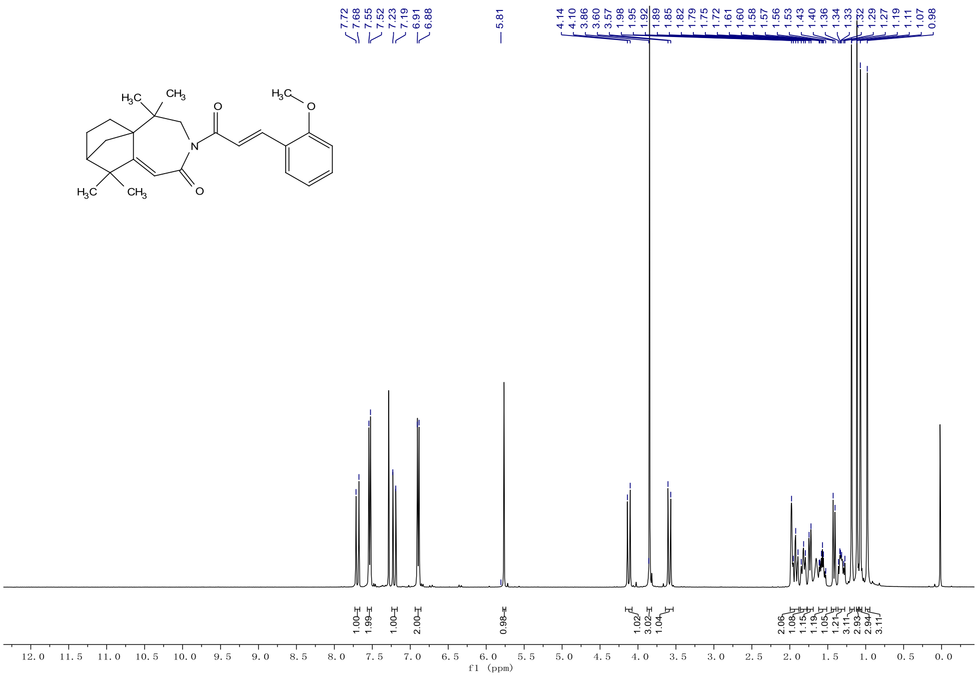

Figure S31: ¹H NMR of compound E16

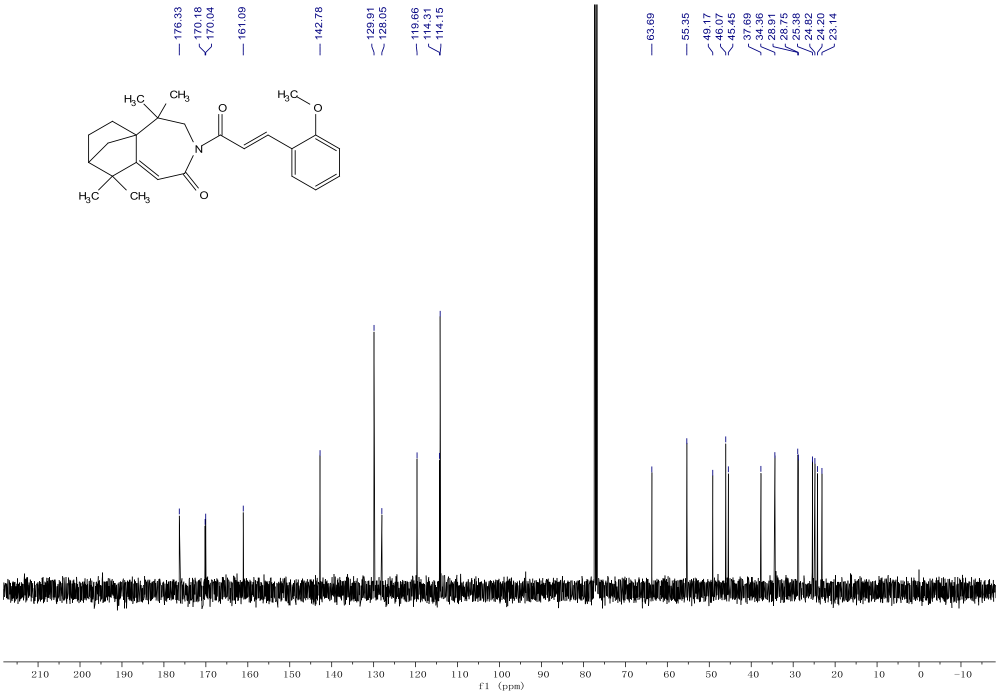

Figure S32: ¹³C NMR of compound E16

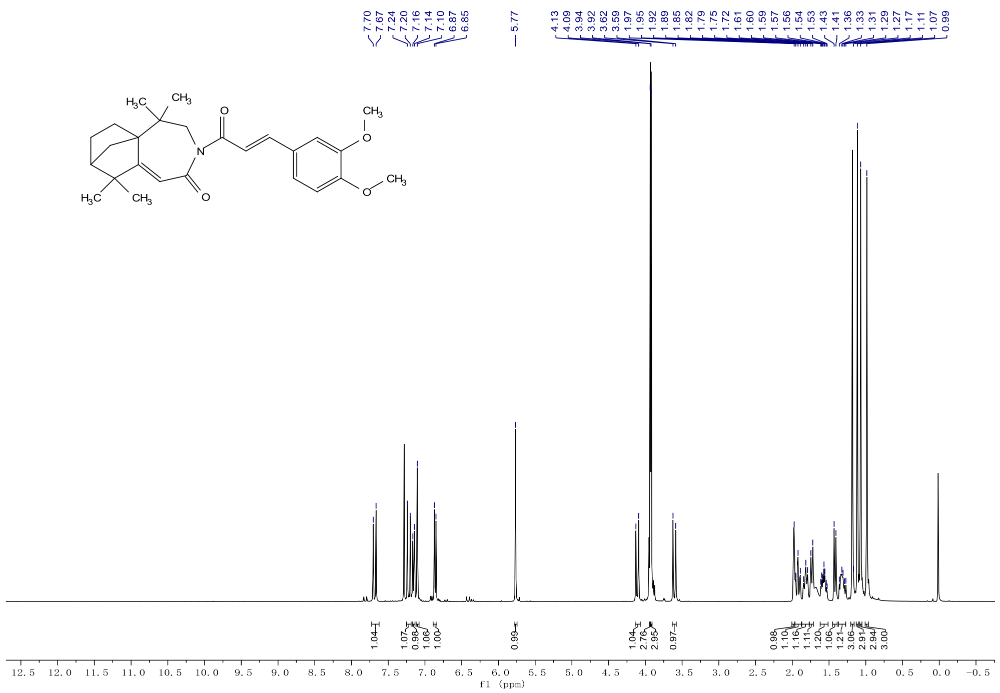

Figure S33: ^{1}H NMR of compound E17

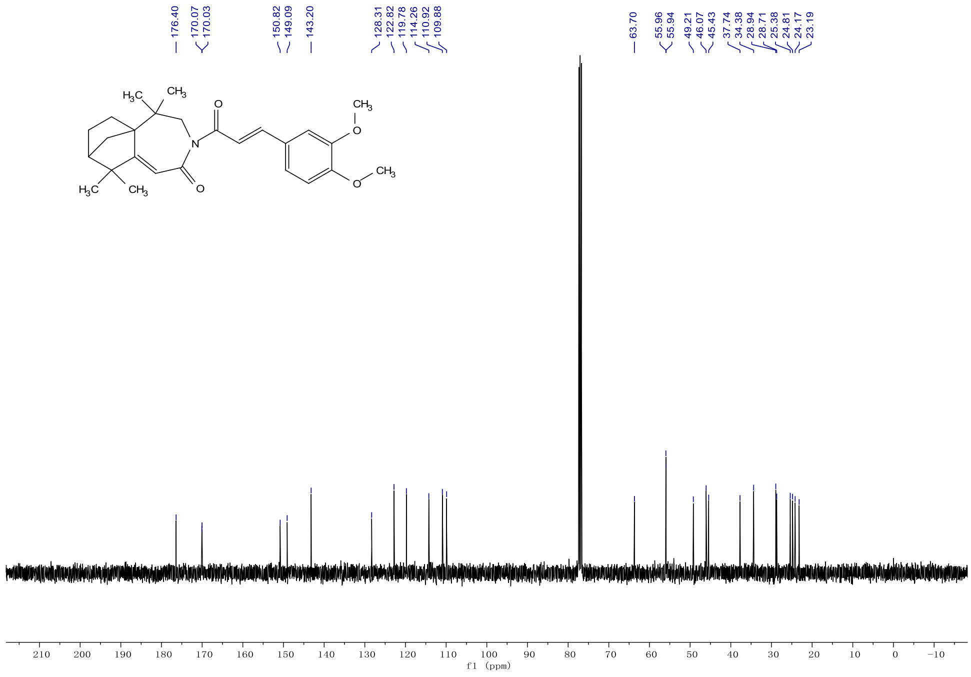

Figure S34: ^{13}C NMR of compound E17

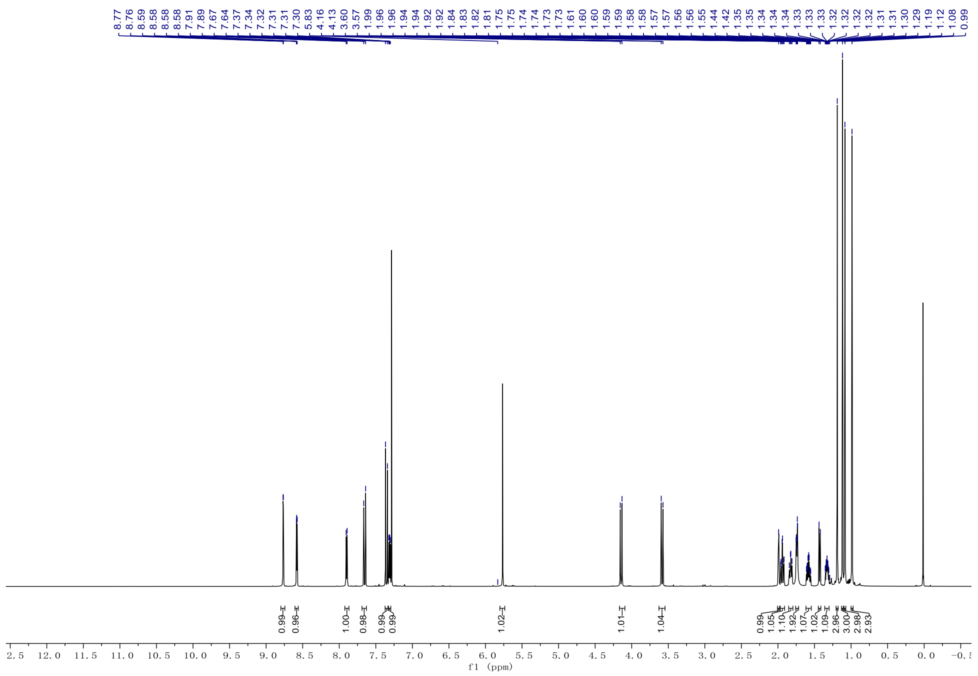

Figure S35: ^{1}H NMR of compound E18

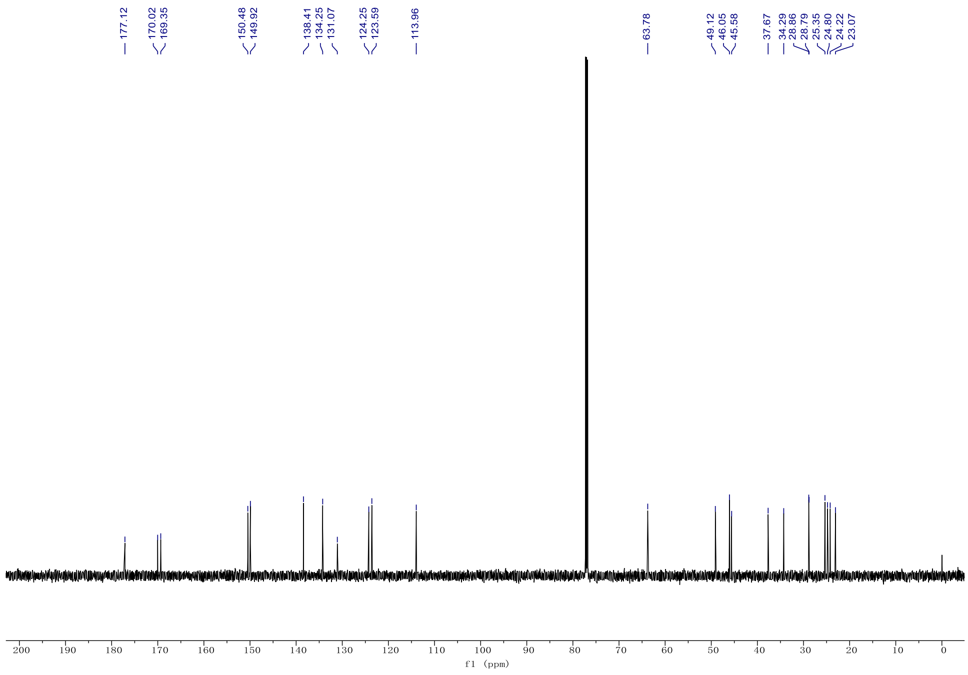

Figure S36: ^{13}C NMR of compound E18

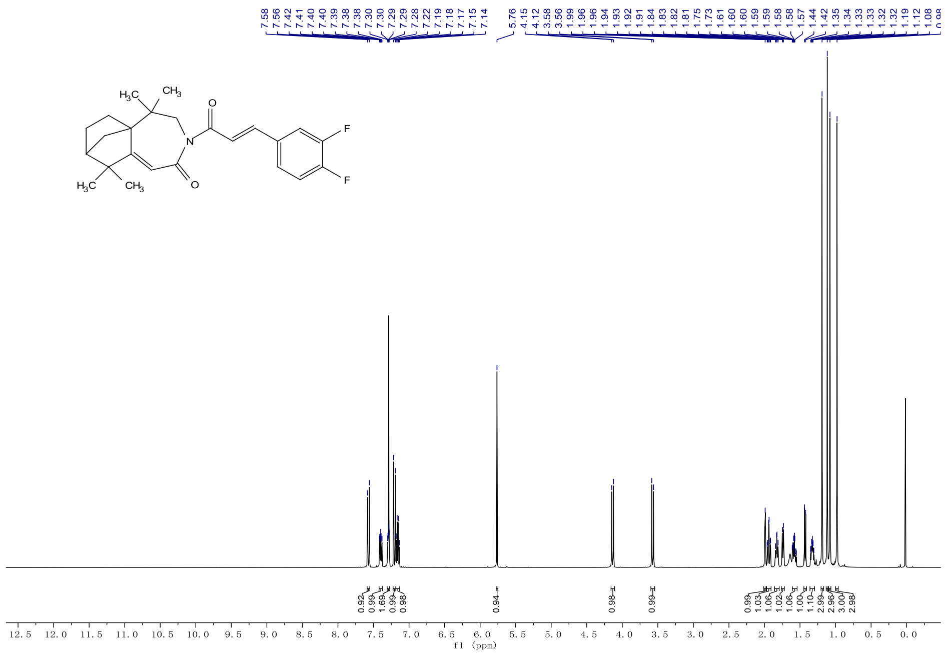

Figure S37: ^{1}H NMR of compound E19

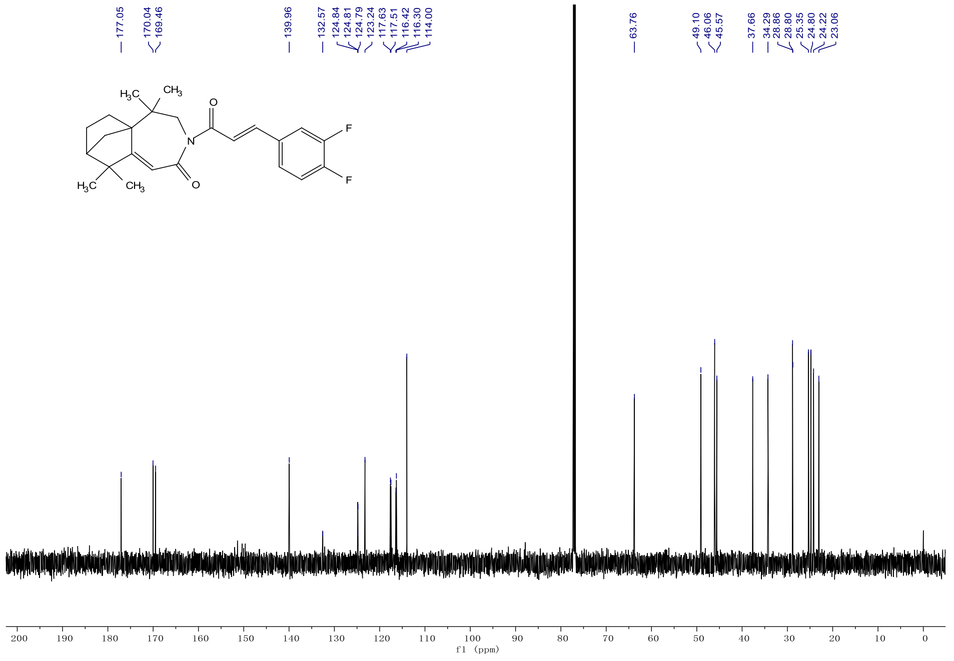

Figure S38: ^{13}C NMR of compound E19

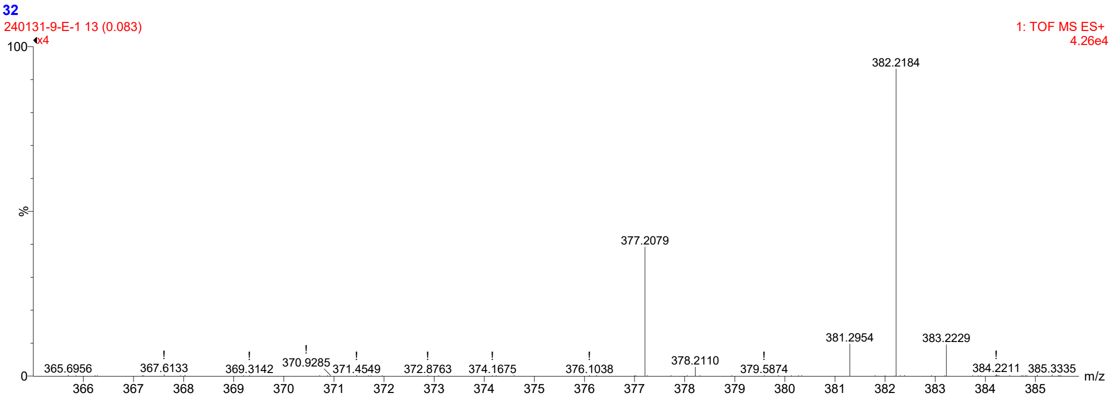

Figure S39: HRMS of compound E1

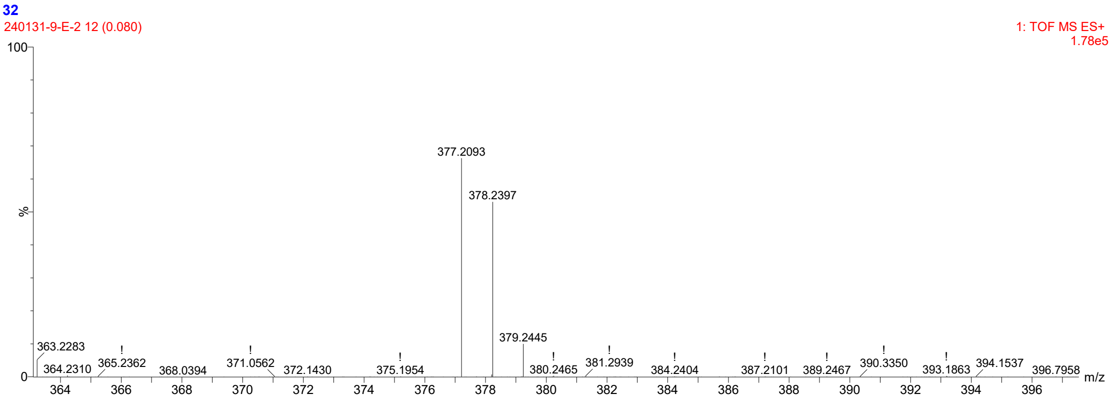

Figure S40: HRMS of compound E2

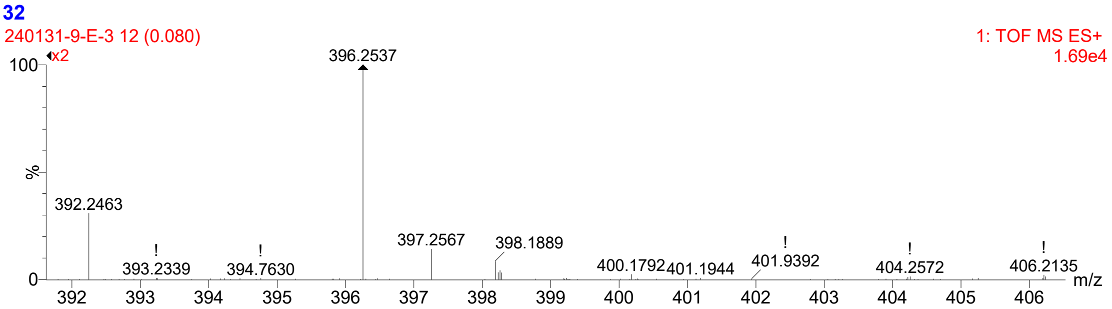

Figure S41: HRMS of compound E3

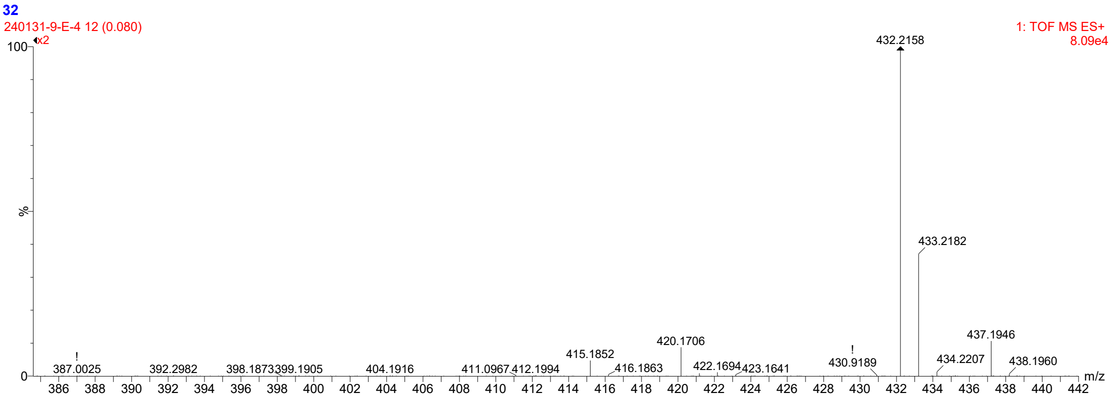

Figure S42: HRMS of compound E4

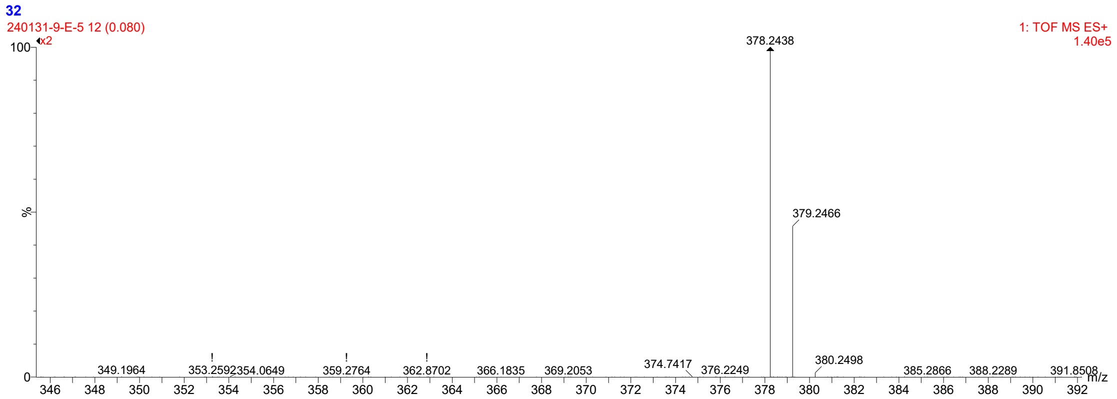

Figure S43: HRMS of compound E5

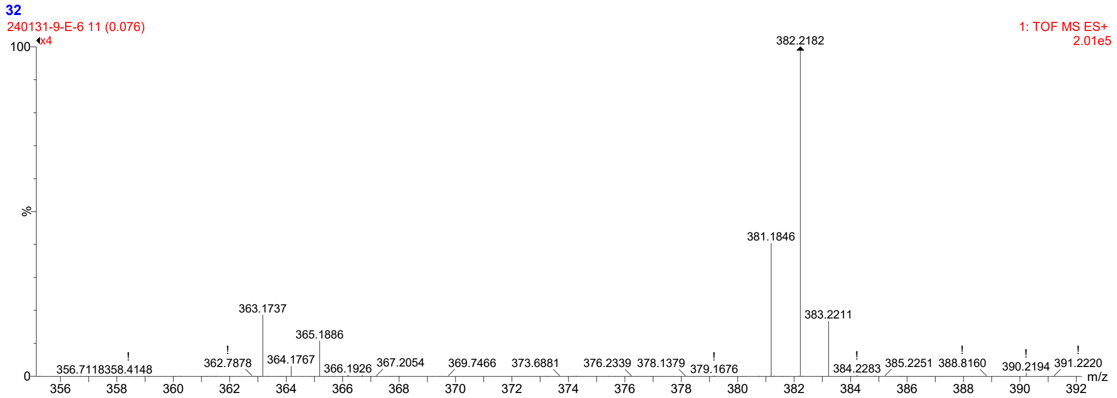

Figure S44: HRMS of compound E6

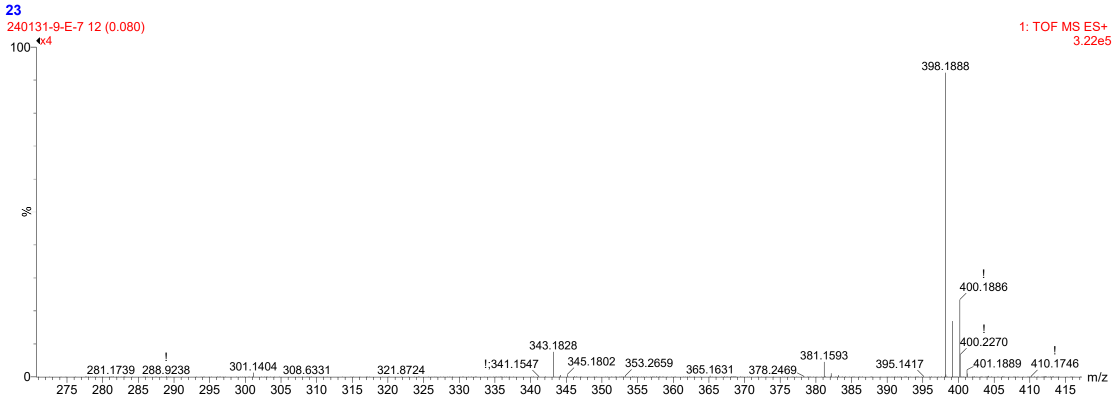

Figure S45: HRMS of compound E7

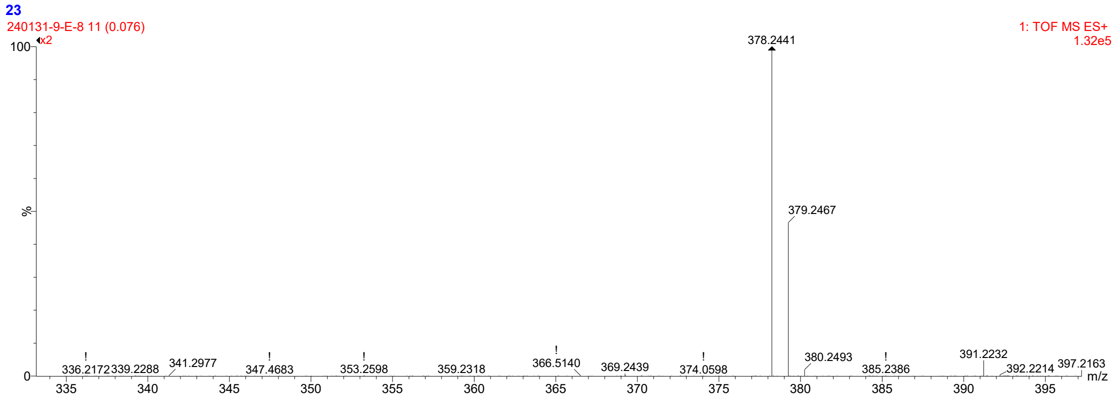

Figure S46: HRMS of compound E8

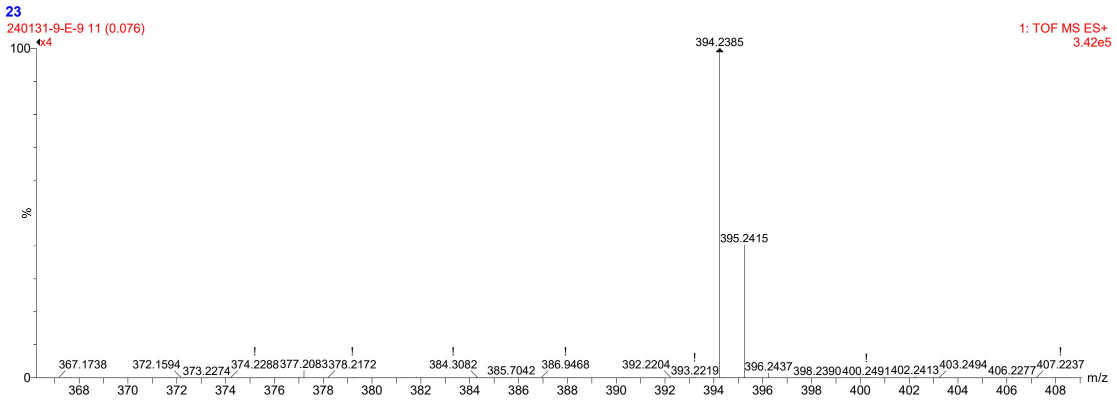

Figure S47: HRMS of compound E9

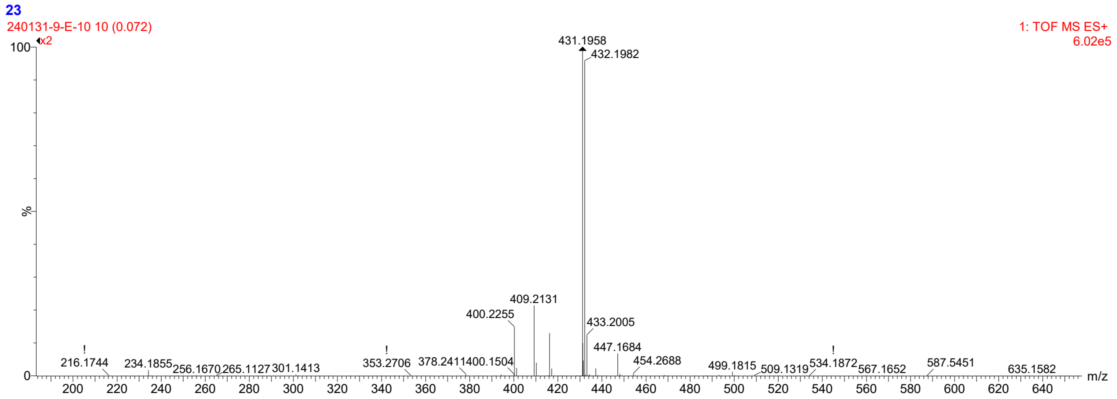

Figure S48: HRMS of compound E10

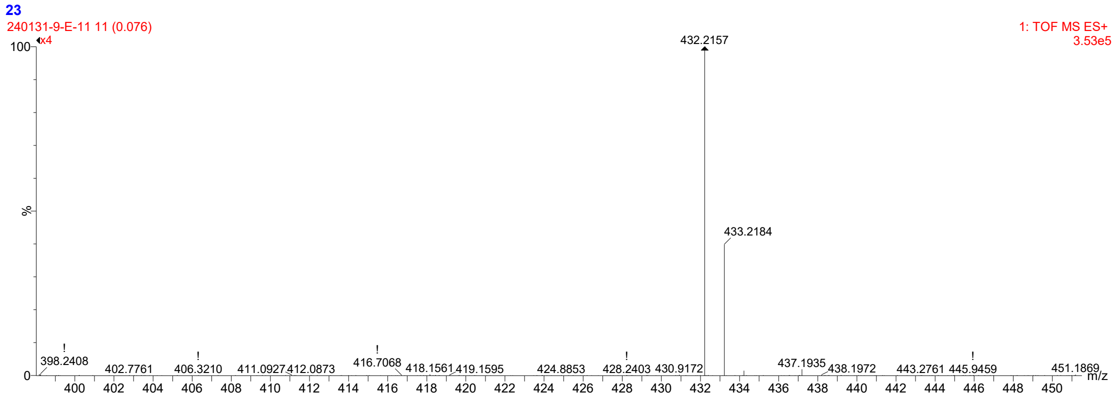

Figure S49: HRMS of compound E11

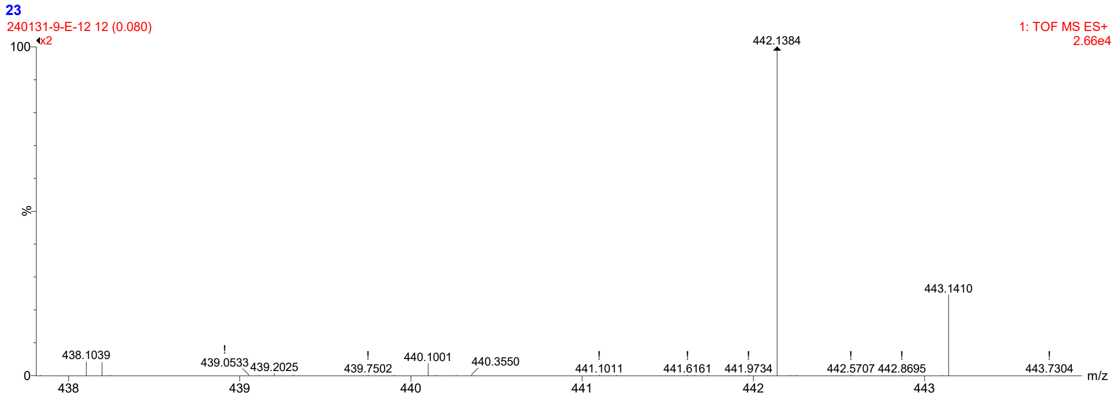

Figure S50: HRMS of compound E12

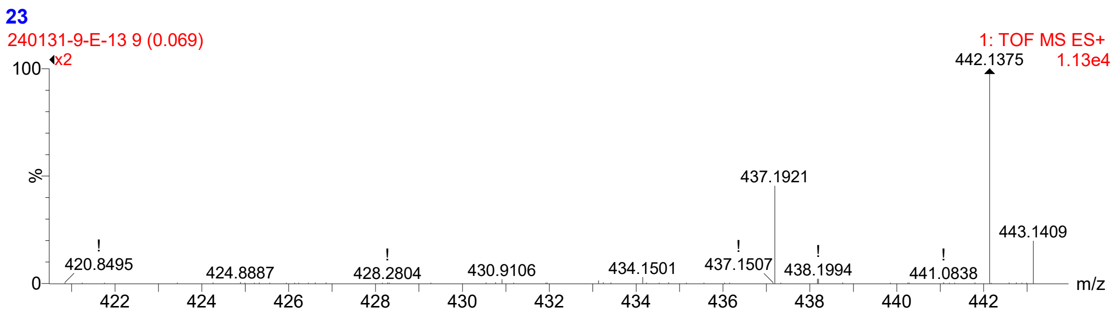

Figure S51: HRMS of compound E13

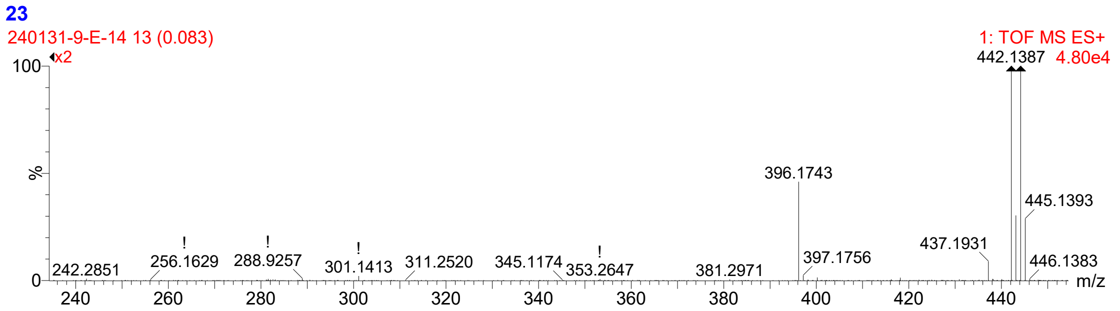

Figure S52: HRMS of compound E14

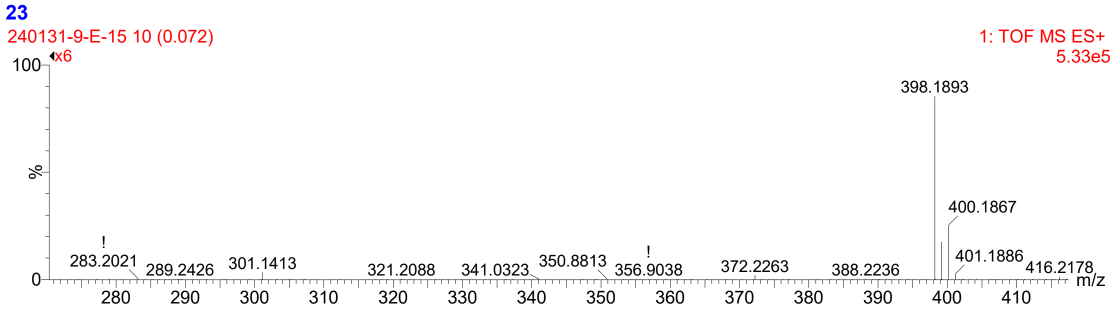

Figure S53: HRMS of compound E15

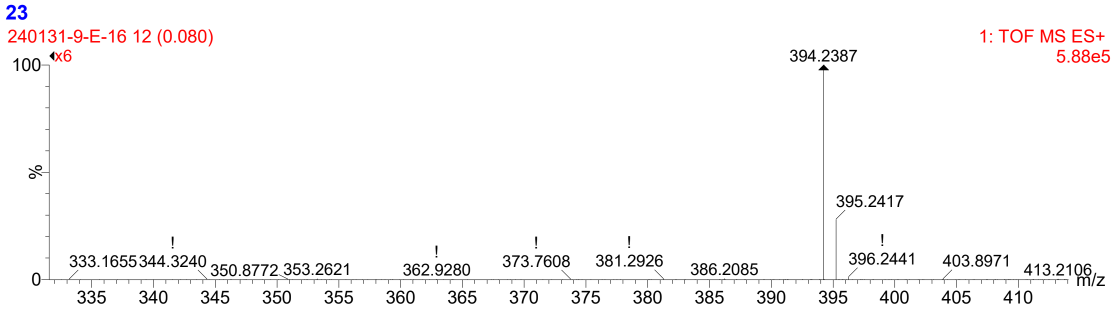

Figure S54: HRMS of compound E16

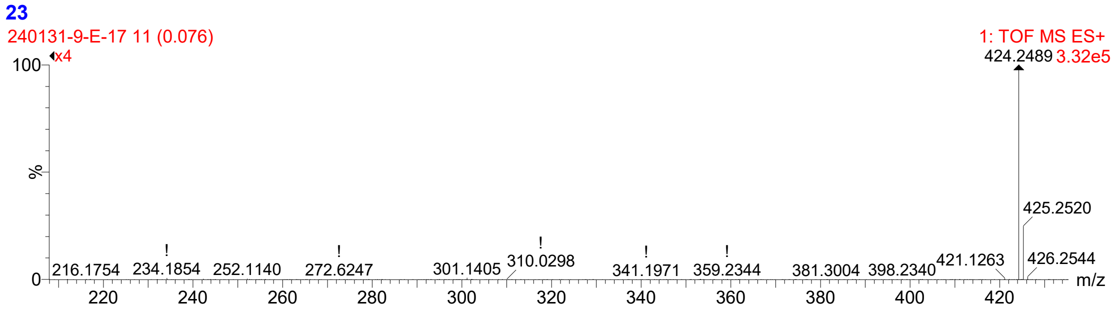

Figure S55: HRMS of compound E17

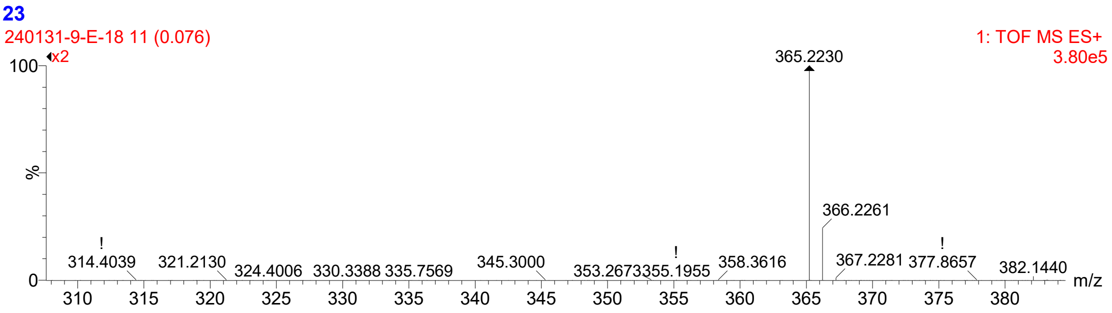

Figure S56: HRMS of compound E18

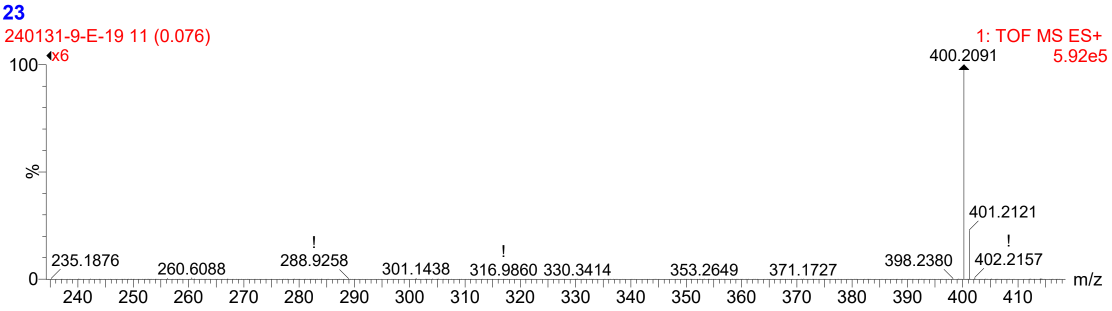

Figure S57: HRMS of compound E19
